# Synthesis and computational evaluation of imidazole-based functional materials for applications in sensing and detection: modulating electronic effects for enhanced performance

**DOI:** 10.1039/d5ra04242a

**Published:** 2025-09-29

**Authors:** Rohini R. Suradkar, Dnyaneshwar P. Gholap, Aarti V. Belambe, Machhindra. K. Lande

**Affiliations:** a Department of Chemistry, Dr Babasaheb Ambedkar Marathwada University Chhatrapati Sambhajinagar Maharashtra India mkl_chem@yahoo.com

## Abstract

Herein, the synthesis of novel organic molecules 2,6-bis-(4,5-diphenyl-1-imidazole-2-yl)pyridine (3A), 2,6-bis-(7*H*-acenaphtho[1,2-*d*]imidazole-8-yl)pyridine (3B) and 2,6-bis-(1*H*-phenanthro[9,10-*d*]imidazole-2-yl)pyridine (3C) was reported. They were synthesized by the Debus–Radziszewski imidazole synthetic method and characterized by FTIR, UV-vis, ^1^H NMR, ^13^C NMR and mass spectrometry. A density functional theory (DFT) approach was used to compute optical analysis, as well as the study of vibrational, frontier molecular orbitals (FMOs) and global indices of reactivity. The electronic transition was explored through the TD-DFT/B3LYP method, which employs time-dependent density functional theory calculations. The recently synthesized compounds were assessed for their fluorescence characteristics, and encouraging findings indicated that the emission efficiency was enhanced through the modulation of conjugation within a molecule. A highly sensitive and selective fluorescent chemosensor exhibited an “on-off” fluorescence response to Fe^3+^ with a 1 : 1 binding ratio in ethanol.

## Abstract 2

Herein, synthesis and fluorescence of novel organic molecules 2,6-bis-(4,5-diphenyl-1-imidazole-2-yl)pyridine (3A), 2,6-bis-(7*H*-acenaphtho[1,2-*d*]imidazole-8-yl)pyridine (3B) and 2,6-bis-(1*H*-phenanthro[9,10-*d*]imidazole-2-yl)pyridine (3C) was reported.

## Graphical abstract

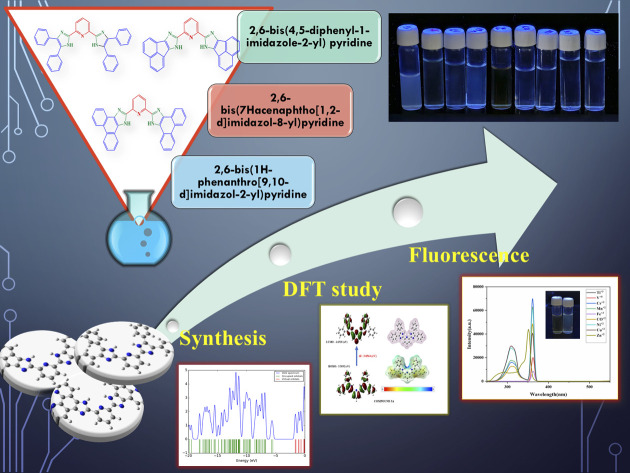

## 1 Introduction

The coordination complexes of transition metals have garnered significant attention lately due to their distinctive photophysical characteristics, including MLCT absorption, comparatively extended excited state lifetimes, and high luminescence competence.^1–3^ The photophysical characteristics of these complexes are influenced by the ligands they are coordinated with and can be readily adjusted through the careful selection and combination of ligands and their substituents. To date, various ligands exhibiting diverse structural and electronic properties have been created and developed to optimize the photophysical traits of these complexes.^4–7^ Among them, the most commonly used ligands are based on 1,10-phenanthroline due to their rigid, planar, hydrophobic, electro-poor heteroaromatic system.^8^ Adding functional groups to phenanthrene or its derivatives allows for fine-tuning of their optical and electrical properties. The fused imidazole ring enhances the molecule's delocalized electron system, improving its light-related characteristics. Furthermore, modifying the ligands with electron-donating or withdrawing groups alters the energy levels of the molecules, directly impacting the light emission of metal complexes containing them. Many metal complexes featuring phenanthrene, such as those with ruthenium, iridium, platinum, rhenium, and copper, have been created and exhibit interesting light-based behaviors.^9–11^ Nonetheless, the electronic configurations and the light-absorbing characteristics of these ligands appear to differ in comparison to phenanthrene^12,13^ and the source of these spectroscopic characteristics has yet to be investigated. Consequently, a comprehensive understanding of the basic photophysical attributes of the ligand with innovative molecular designs is needed to better inform the future molecular development of new complexes. The examination of the structural and spectroscopic traits of different molecules through both experimental and theoretical approaches has captured the attention of scientists for numerous years. In particular, density functional theory (DFT) calculations have proven valuable for investigating molecular characteristics, including structural, spectroscopic, and photophysical features.^14–16^ Due to their applications in biology, environmental monitoring, and industry, there's been a surge of interest in creating highly selective and sensitive sensors for detecting heavy and transition metal ions. Among a variety of metal ions, iron stands out as the most prevalent and vital transition element in the human body, contributing significantly to physiological functions such as electron transfer, oxygen absorption, gene regulation, and oxygen metabolism. An overload or lack of iron can lead to a wide range of health issues.^17^ Furthermore, contamination of water and soil by metal ions such as iron, copper, and others has garnered considerable attention due to the risks it poses to our everyday lives. Therefore, it is crucial to identify and measure these metal ions in both biological and environmental contexts. Present techniques for detecting Fe^3+^, including atomic absorption spectroscopy, mass spectrometry, and electrochemical spectroscopic analysis, face challenges due to their complex and intricate nature, or the lengthy duration of experiments. Conversely, fluorescence methods are gaining popularity because of their ease of use and exceptional sensitivity.^18,19^

Based on our literature search, no existing studies have used quantum chemical analysis to examine the reactivity, charge distribution, or geometric and spectroscopic properties of the synthesized molecule. The main aim of the present research is to synthesize compounds and comparative studies through experimental and computational analysis of various parameters and their importance in metal ion detection.

The use of B3LYP/6-311G(d,p) for DFT and TD-DFT calculations provides a consistent framework, but its effectiveness in accurately describing excited-state properties raises concerns. While B3LYP is reliable for ground-state geometries, it struggles with charge-transfer excitations and predicting *λ*_max_ values for systems with significant delocalization due to its lack of long-range correction. To enhance accuracy and gain deeper insights into photophysical properties, exploring more advanced functionals like M06-2X, CAM-B3LYP, or ωB97XD, which better address delocalization errors and long-range interactions, is essential. Although discussing B3LYP's limitations and conducting preliminary comparisons with these functionals would strengthen the computational methodology.

The current research also aims to refine chemical concepts related to molecular structure by optimizing parameters using efficient and cost-effective computational methods instead of expensive and labor-intensive experimental approaches.^20,21^ In this manuscript, we present a design and synthesis of a novel molecule synthesized by the Debus–Radziszewski imidazole synthesis method, and characterized by ^1^H NMR ^13^C NMR, GC-MS the definitive structure of the molecule was validated using powerful analytical instruments and computational analysis.^22^ DFT was used to computationally analyze the molecule, revealing their reactivity and active sites.^23^ This study is highly valuable for metal ion detection. DFT-based simulations of IR and UV-vis spectra enhance its practical application in experimental research, providing a powerful tool for data analysis. The reason for conducting this study is that it yields accurate results for compounds while maintaining a low computational expense. Computational analyses have been performed using DFT/B3LYP/6-311G basis sets at the ground-state level. To explore molecular structures, computational researchers calculated quantum chemical descriptors. Additionally, TD-DFT was used to simulate the UV-vis spectra of each compound.^24^ Spectroscopic characteristics have additionally been explored by refining their FT-IR vibrational parameters using different basis sets.^25–27^ This study investigates the photophysical properties of three compounds containing benzil, acenaphthoquinone, and phenanthroline groups by analyzing their optical absorption and photoluminescence. Particular attention is paid to comparing the calculated structural and spectral properties of these ligands with experimental findings.

## 2 Experimental section

### 2.1 General information

All chemicals and solvents were sourced from Sigma-Aldrich, Merck, and Molychem, ensuring high purity, and were utilized directly in the reaction process without any additional purification. The advancement of all organic transformations was initially monitored using thin-layer chromatography (TLC). ^1^H NMR spectra (500 MHz) and ^13^C NMR spectra (500 MHz) were conducted on an Avance-II (Bruker) FT NMR spectrometer model, using DMSO-*d*_6_ solvent and TMS as the internal standard. FTIR spectra were obtained from Shimadzu FTIR 8300 spectrophotometer. Mass spectra were collected using a Waters Micromass Q-Tof Micro, with electrospray ionization (ESI) and atmospheric pressure chemical ionization sources, capable of analyzing a mass range of 4000 amu in quadrupole mode and 20 000 amu in ToF mode. The fluorescence of the compound was evaluated using a spectrofluorometer from SHIMADZU corp 00 643.

### 2.2 Procedure for synthesis of 2,6-bis(4,5-diphenyl-1-imidazole-2-yl) pyridine (3A)

**Scheme 1:**
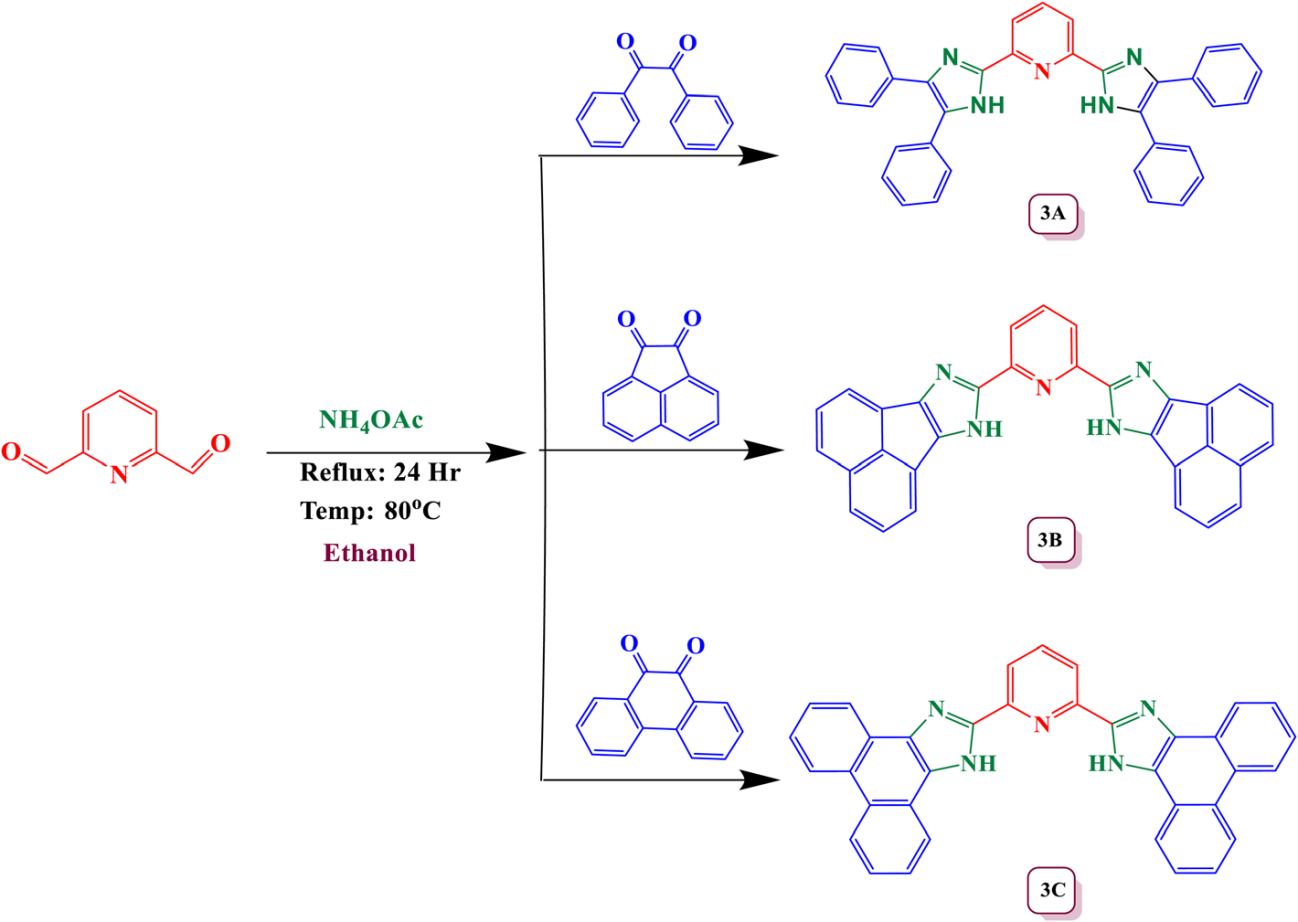
: Synthetic pathway of imidazole-based ligand by using Debus–Radziszewski synthesis method.

2,6-bis(4,5-diphenyl-1-imidazole-2-yl) pyridine the compound was synthesized using the Debus–Radziszewski imidazole synthesis method through a direct reaction between pyridine-2,6-dicarbaldehyde (1 mmol), ammonium acetate (4 mmol) and benzil (2 mmol) was dissolved in 15 ml of ethanol and refluxed with stirring at 80 °C for 24 h (depicted in Scheme 1). The advancement and finalization of the reaction were observed using thin-layer chromatography (TLC). Once the reaction was finished, the mixture was poured into ice-cold water, leading to the creation of a precipitate. To achieve a pure crystalline product, the residue was further subjected to recrystallization using ethanol as the solvent. All the experimental spectra are shown in the SI (Spectrum 1–4).

Yield: 76.80%.

Melting point: 118–120 °C.

FTIR (*υ*_max_ cm^−1^): 3574.26(N–H), 1422.98(C–N), 1582.88 (C=N).

^1^H NMR (500 MHz, DMSO *δ* ppm): 8.35 (s, 1H), 7.93–7.92 (m, 10H), 7.82 (t, *J* = 8.5 Hz, 1H), 7.79 (d, *J* = 8.2 Hz, 2H), 7.64–7.62 (m, 10H).

^13^C NMR (500 MHz, DMSO *δ* ppm): 194.7, 135.4, 132.1, 129.5, 129.4.

Mass C_33_H_20_N_8_O, M^+^: 516.21.

### 2.3 Procedure for synthesis of 2,6-bis(7*H*-acenaphtho[1,2-*d*]imidazole-8-yl)pyridine (3B)

2,6-bis(7*H*-acenaphtho[1,2-*d*]imidazole-8-yl)pyridine was synthesized *via* Debus–Radziszewski imidazole method by refluxing pyridine-2,6-dicarbaldehyde (1 mmol), ammonium acetate (4 mmol) and acenaphthoquinone (2 mmol) in 15 ml of ethanol with constant stirring at 80 °C temperature for 24 h (depicted in Scheme 1). TLC was used to monitor the reaction's progress and confirm its completion. At the end of the reaction, the mixture was poured into ice and the solid that appeared was purified by recrystallization with hot ethanol. The SI file contains all the experimental spectra. (Spectrum 5–8)

Yield: 70.16%.

Melting point: 133–135 °C.

FTIR (*υ*_max_ cm^−1^): 3746.19(N–H), 1582.52(C–N), 1497.52(C=N).

^1^H NMR (500 MHz, DMSO *δ* ppm): 8.55 (s, 1H), 8.45–8.43 (m, 3H), 8.09–8.07 (m, 6H), 7.94–7.92 (m, 6H).

^13^C NMR (500 MHz, DMSO *δ* ppm): 187.5, 144.2, 135.5, 132.2, 130.4, 128.8, 128.4, 121.1.

Mass C_33_H_20_N_8_O, M^+^: 459.13.

### 2.4 Procedure for synthesis of 2,6-bis(1*H*-phenanthro[9,10-*d*]imidazole-2-yl)pyridine (3C)

2,6-bis(1*H*-phenanthro[9,10-*d*]imidazole-2-yl)pyridine was synthesized *via* Debus–Radziszewski imidazole synthetic method by refluxing pyridine-2,6-dicarbaldehyde (1 mmol), ammonium acetate (4 mmol) and phenanthroline (2 mmol) in 15 ml of ethyl alcohol with constant stirring at 80 °C temperature for 24 h (depicted in Scheme 1). Thin-layer chromatography (TLC) was used to track the reaction's progression and confirm its completion. Once finished, the reaction mixture was poured into crushed ice. The resulting solid was then collected by filtration and purified through recrystallization using hot ethanol. All the experimental data is included in the SI file (Spectrum 9–12).

Yield: 69.20%.

Melting point: 148–150 °C.

FTIR (*υ*_max_ cm^−1^): 3756.66(N–H), 1583.11(C–N), 1440.52(C=N).

^1^H NMR (500 MH_Z_, DMSO *δ* ppm): 8.30 (s, 3H), 8.03–8.02 (m, 4H), 7.88–7.77 (m, 6H), 7.55–7.52 (m, 6H).

^13^C NMR – (500 MHz, DMSO *δ* ppm): 178.9, 135.4, 135.2, 131.1, 129.2, 129.0, 124.3.

Mass C_33_H_20_N_8_O, M^+^: 512.15.

### 2.5 Computational section

To calculate the quantum chemical parameter of imidazole derivatives Gaussian 09,^28^ GuassView 5.0,^29^ and ChemDraw version (15.0) programs were used often. DFT calculations, specifically B3LYP/6-311G(d,p), were used to determine geometric parameters. Molecular geometries were optimized with various Gaussian basis sets to ensure accurate energy minima. Compound 3A is considered a reference molecule and the optimization was performed using two distinct basis sets, 6-31G(d,p) and 6-311G(d,p), yielding optimization energies of −1622.6447 and −1622.9826 Hartree, respectively. The basis set 6-311G(d,p) was sensibly selected based on it having given minimum optimization energy −1622.9826 Hartree. All these calculations were done in the gas phase. A range of quantum chemical characteristics such as the energy of the lowest unoccupied molecular orbital (*E*_LUMO_), the energy of the highest occupied molecular orbital (*E*_HOMO_), and the energy gap (EGAP) have been examined. In addition, values for absolute softness (*σ*), absolute hardness (*η*), absolute electronegativity (*χ*), electrophilicity index (*ω*), chemical potential (CP), dipole moment (*μ*), and global softness (*S*) have been determined.^30^

## 3 Results and discussion

### 3.1 Molecular geometry

**Fig. 1:**
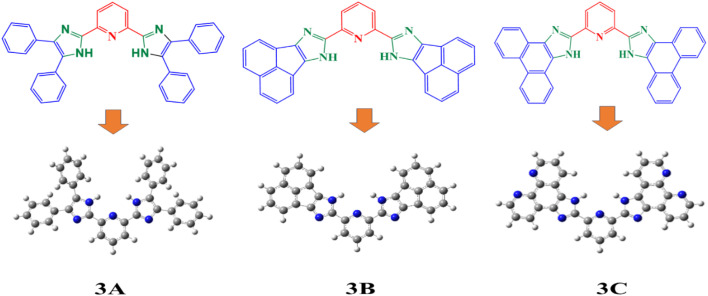
The optimized theoretical geometric structures of the synthesized derivates at DFT/B3LYP/6-311G(d,p).

The arrangement of newly created imidazole derivatives was refined utilizing DFT at the B3LYP theoretical level along with the 6-311G(d,p) basis set to examine the geometric characteristics. The optimized structures and geometrical parameters of imidazole derivatives each of the molecules, 3A, 3B and 3C, are within the C_1_ point group and contain the following degrees of freedom: 189, 153 and 165. The bond characteristics, including bond lengths and bond angles of the created compound, were refined, and the refined structure of the synthesized derivatives is illustrated in Fig. 1. The C=N bond lengths are 1.3195 Å for the 3A molecule and 1.3307 Å for 3B and also for 3C 1.3204 Å, which are observed and are in agreement with the reported value.^31,32^

These synthesized compounds display behavior indicative of double bonds. However, C–N bond lengths are observed at 1.3818 Å for 3A and 1.3805 Å for 3B, and 3C bond lengths are observed at 1.3720 Å. The observed C–N bond lengths are shorter than typical single bonds, indicating resonance effects within the region of the molecule.^33^ The N–H bond lengths observed for 3A, 3B and 3C are 1.008, 1.0073 and 1.0069 Å respectively. The variations in bond length and bond angle within the derivatives can be attributed to the existence of intermolecular interactions, alongside lone pair electrons, electronegativity, and conjugation, which significantly influence the molecular framework. As the conjugation increases, a shorter bond length is observed in N–H bonds. The selected bond angles and bond lengths of all optimized molecules are shown in Table 1.

Selected geometric parameter of synthesized derivatives calculated at DFT/B3LYP/6-311G(d,p)

**Table:** 

3A		3B		3C	
Bond length (Å)		Bond length (Å)		Bond length (Å)	
9C–12N	1.3618	10C–50N	1.3805	47C–50N	1.3720
9C–11N	1.3195	10C–51N	1.3307	47C–51N	1.3204
12N–64H	1.0080	50N–11H	1.0073	50N–57H	1.0069
10C–13N	1.3618	13C–49H	1.3805	28C–48N	1.3720
10C–14N	1.3195	13C–48C	1.3307	28C–49N	1.3204
13N–65H	1.0080	49N–14H	1.0073	48N–56H	1.0069
5C–15N	1.3435	1C–6N	1.3446	24N–23C	1.3433
1C–15N	1.3435	5C–6N	1.3446	24N–19C	1.3433

**Table:** 

Bond angle (°)		Bond angle (°)		Bond angle (°)	
5C,9C,12N	122.4747	2C,1C,6N	122.9008	20C,19C,24N	122.9967
5C,9C,11N	126.7058	4C,5C,6N	122.9008	22C,23C,24N	122.9963
4C,5C,15N	122.9302	1C,10C,50N	121.6378	19C,47C,50N	121.8955
2C,1C,15N	122.9302	1C,10C,51N	126.2930	19C,47C,51N	126.1542
14N,10C,13N	110.8191	50N,10C,51N	112.0692	51N,47C,50N	111.9504
10C,13N,65H	124.1463	10C,50N,11H	123.0698	47C,50N,57H	123.4681

### 3.2 FT-IR vibrational analysis

Gaussian09 has been utilized for IR-vibrational computations, and their representation has been enhanced with animation features. All frequencies calculation is performed on 6-311G(d,p) basis set. Compound I (3A), which consists of 65 atoms, exhibits 189 vibrational modes, whereas compound II (3B), with 53 atoms, demonstrates 153 fundamental vibrational modes. Compound III (3C), containing 57 atoms, has revealed 165 vibrations, adhering to the IR vibrational 3N-6 rule. The IR spectra for these compounds, obtained through DFT, are presented in Table 2, and the computed frequencies have been compared with their corresponding experimental measurements. All vibrational modes are listed in order from the highest to the lowest wavenumber values.^34^ In all three compounds, major vibrations stretching like rocking, and scissoring as well as a symmetric and asymmetric mode of the band were seen. All the theoretical vibrational spectrum is included in the SI file. (Spectrum 13–15)

**Table 2:** Experimental and simulated IR spectra of a synthesized molecule at DFT/B3LYP/6-311G(d,p) basis set

Assignment	3A		3B		3C	
	Experimental (cm^−1^)	Theoretical (cm^−1^)	Experimental (cm^−1^)	Theoretical (cm^−1^)	Experimental (cm^−1^)	Theoretical (cm^−1^)
N–H	3574.26	3648.24	3746.19	3658.21	3756.66	3661.13
C–N	1422.98	1448.07	1582.52	1512.86	1583.11	1575.56
C=N	1582.88	1590.07	1497.52	1496.10	1440.52	1437.10

#### 3.2.1 N–H stretching

The N–H stretching vibrations stand in the region of IR 3366 and 3710 cm^−1^ all three synthesized compounds have two NH groups.^35^ while the theoretical NH stretching in the 3A, 3B, and 3C compound is observed at 3648.24 cm^−1^, 3658.21 cm^−1^, 3661.13 cm^−1^ with symmetric stretching respectively. Also, the experimental value is observed for 3A, 3B and 3C at 3574.26 cm^−1^, 3746.19 cm^−1^ and 3756.66 cm^−1^ respectively.

#### 3.2.2 C–N and C=N vibrations

Determining the C=N and C–N vibrations is challenging because the area allows for the combination of multiple bands.^36^ The C=N and C–N stretching frequencies observed at 1590–1690 cm^−1^ and 1200–1450 cm^−1^ in the FT-IR spectrum, respectively^37^ In this current research, a C=N stretching vibration is noted for compounds 3A, 3B and 3C at 1590.07, 1575.18 and 1543.48 cm^−1^, respectively. Also, C–N stretching vibrations are observed at 1448.07 cm^−1^, 1575.18 cm^−1^ and 1543.48 cm^−1^, respectively.

The DFT computations serve as an effective method for distinguishing between C–N (single bond) and C=N (double bond) vibrational bands by examining their symmetric and asymmetric stretching modes. Owing to variations in bond order, length, and strength, C–N and C=N bonds display unique vibrational frequencies. In a molecule featuring C–N or C=N groups, these computations can uncover both symmetric and asymmetric stretching vibrations. Typically, asymmetric stretching occurs at higher frequencies compared to symmetric stretching for a particular bond type. By visualizing the displacement vectors for each calculated vibrational mode, one can accurately associate a computed frequency with a specific C–N or C=N stretching motion and further differentiate between its symmetric and asymmetric variations.

The mean absolute deviations for all computed properties compared to the experimental data gathered using the DFT/B3LYP/6-311G(d,p) basis set are detailed in the SI file (Fig. 11). A remarkable correlation between theoretical predictions and experimental findings is confirmed for approximately 75% of the results within a specified deviation range. The most significant mean absolute deviations were noted for the NH in relation to the experimental data.

The determined frequencies were contrasted with their corresponding experimental values and demonstrated a strong correlation between the two. The calculated values exhibited a close alignment with the related experimental figures, while the discrepancies can be attributed to phase transitions between the calculated and experimental vibrational frequencies.

### 3.3 Frontiers molecular orbitals and MEP analysis

**Fig. 2:**
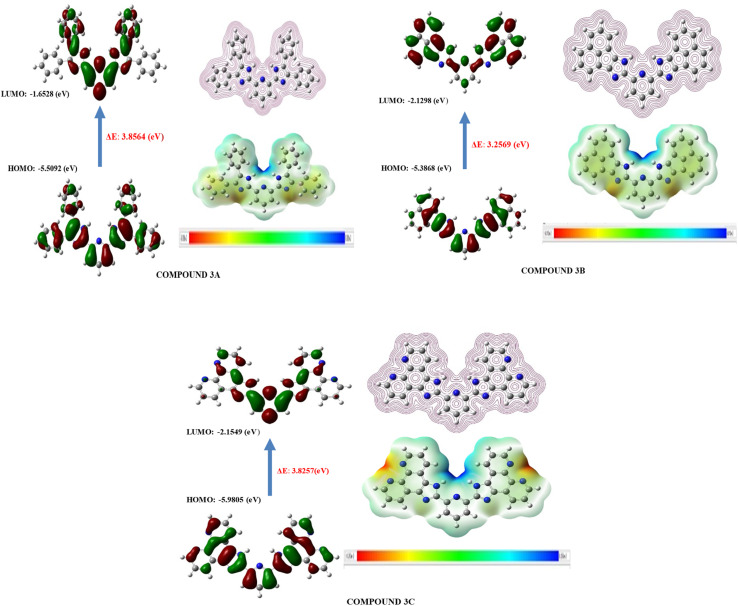
FMO's, molecular electrostatic potential and contour image of 3A, 3B and 3C calculated at DFT/B3LYP/6-311G(d,p).

HOMO and LUMO orbitals, crucial in quantum chemistry, determine chemical stability and reactivity. The HOMO represents electron donation, and the LUMO, electron acceptance. These orbitals influence electrical properties and intermolecular interactions. The synthesized molecular structure and theoretically optimized structures of molecules were calculated at the DFT/B3LYP/6-311G basis set, as shown in Fig. 1. The HOMO and LUMO are the primary orbitals involved in chemical reactions. The HOMO energy correlates with ionization potential and the LUMO energy with electron affinity. These frontier orbitals can predict adsorption sites for inhibitor molecules on metal surfaces, which is crucial in corrosion prevention.^38^ The graphical presentation of HOMO and LUMO energies and the band gap of the optimized molecules is shown in Fig. 2. The band gap energy value of synthesized derivatives 3A, 3B and 3C was found to be 3.8564, 3.2569 and 3.8257 eV respectively, in the gas phase. According to recommended computational parameters, a high HOMO energy suggests that the compound readily donates electrons, thus increasing its reactivity or biological activity towards electron acceptors.^39^

Based on the *E*_HOMO_ values, the compound reactivity trend will follow compound 3B > compound 3A > compound 3C. Although lower *E*_LUMO_ values suggest that compounds have strong electron-accepting abilities and act as reactive entities for electron-donating compounds, the reactivity follows the trend in this sequence, compound 3B > compound 3C > compound 3A. Also, the energy band gap of the synthesized compound follows the order 3A (3.8564) > 3C (3.8257) > 3B (3.2569). When π-orbitals extensively interact throughout a molecule, they create delocalized molecular orbitals. As the degree of π-conjugation rises, the number of molecular orbitals also increases, and the energy differences between them typically diminish. The 3B molecule is likely to exhibit the most extensive and effective π-conjugation, featuring a distinctive five-membered ring linked to a naphthalene unit, which imparts a slightly strained and non-planar nature in contrast to entirely planar structures. The existence of the double bond within the five-membered ring can greatly influence conjugation. The 3C molecule demonstrates high levels of conjugation, likely enhancing efficient π-delocalization and resulting in a potentially narrower gap when compared to systems with less extensive conjugation, whereas the 3A molecule presumably showcases the least efficient π-conjugation. This may stem from the fact that the conjugation in benzil is somewhat disrupted by the single bonds linking the phenyl rings, which allows for a degree of rotational flexibility.

As shown in the SI file (Table 5), the difference between frontier molecular orbitals not only indicates the stability of the molecule but is also connected to its interactions with other entities. Smaller energy differences lead to softer molecules, which are associated with higher reactivity and reduced kinetic stability, thus enhancing the fluorescence activity of the compounds as the energy gaps decrease.

#### 3.3.1 Molecular electrostatic potential

The MEP description assists in comprehending the connections both within and between molecules, such as hydrogen bonding. It identifies electrophilic and nucleophilic interactions and clarifies biological interactions like drug–protein binding. Additionally, it demonstrated how molecules behave and respond to binding locations within the biological structure.^40^ The MEP and contour image of synthesized molecules calculated using DFT/B3LYP with the 6-311G(d,p) basis set is illustrated in Fig. 2, derived from the SCF energy. This visualization serves to evaluate molecular polarity; the areas coloured in blue (positive) represent the sites where nucleophiles can attack, whereas the yellow and red (negative) sites prone to electrophilic attack are shown by these regions. Hydrogen atoms that are bonded to highly electronegative elements are represented by more intense blue hues, whereas nitrogen groups are shown in red and orange, and neutral benzene ring atoms are light green on the MEP surface. The electrostatic potential increases in this order:

Red < orange < yellow < green < blue.

The presence of a blue hue localized around the hydrogen atoms of imidazole rings contributes to the optimal radical scavenging activity.^42,43^ All three molecules' electrostatic potential values were assessed (shown in Table 6 in the SI file), revealing that the reactivity order of the compounds exhibits a specific trend.

Compound 3C > compound 3B > compound 3A.

### 3.4 Global reactivity parameter

The LUMO, HOMO, and Δ*E* from FMO analysis were used to calculate global reactivity parameters (GRPs), revealing the compound's charge transfer, stability, and reactivity. DFT with B3LYP/6-311G(d,p) and specific formulas were used for these calculations.^41^*I* = −*E*_HOMO_*A* = −*E*_LUMO_*E*_GAP_ = *E*_LUMO_ − *E*_HOMO_*η* = (*I* − *A*)/2
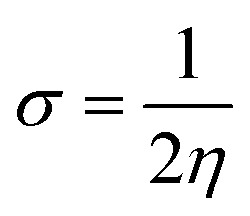
*χ* = (*I* − *A*)/2

The trend in reactivity for the computed values of compounds should also be considered as an additional characteristic linked to FMOs (Fig. 4); this includes chemical hardness and softness, which are inversely related. A chemical hardness of zero indicates the highest level of chemical softness.^42,43^ In this scenario, the 3B compound is less rigid in comparison to other molecules, and this characteristic can be understood through the HSAB theory, which states that ‘‘Hard molecules possess greater energy gaps than soft molecules.’’ Since biological systems are made up of soft cells and enzymes, they are more likely to interact and bond with soft molecules rather than with those that are harder.^44,45^ The substances that exhibit greater dipole moment values (*μ*) indicate longer bond lengths and enhanced charge distribution, making them more localized molecules or more electrophilic systems. This characteristic is associated with increased conductivity during oxidation processes. Conversely, molecules with lower dipole moments tend to accept a smaller amount of electronic charge compared to those with higher dipole moments.

Compound 3A > compound 3B > compound 3C.

The global reactivity parameters are linked to the fluorescence behavior of the molecule and are clarified through Koopman's theorem, which offers a practical and swift method as illustrated in Table 3.^46^

**Table 3:** Calculation of global reactivity Parameters for titled compounds computed at DFT/B3LYP/6-311G(d,p)

Global reactivity descriptors	Compound 3A	Compound 3B	Compound 3C
Optimization energy (Hartree)	−1622.9826	−1465.6627	−1684.7703
HOMO (eV)	−5.5092	−5.3868	−5.9805
LUMO (eV)	−1.6528	−2.1298	−2.1549
Band gap (eV)	3.8564	3.2569	3.8257
Ionization potential (eV)	5.5092	5.3868	5.9805
Electron affinity (eV)	1.6528	2.1298	2.1549
Absolute hardness (*η*)	1.9282	1.6285	1.9128
Absolute softness (*σ*)	0.9641	0.8142	0.9564
Absolute electronegativity (*χ*)	3.5810	3.7583	4.0677

Higher electronegativity and reduced chemical potential values enhance electronic transfer since compounds with delocalized electron clouds can readily interact with other molecules. Therefore, the ranking of activity should be compound 3C > compound 3B > compound 3A conclusively, we could say that high electronegativity is directly proportional to emission, and low chemical potential is also directly proportional to emission. The energy gap between the valence and conduction bands increases leading to a reduction in the emission intensity however, variations can also occur due to the reduced conjugation in the molecules. This study showed that the molecule possessed a greater number of functional groups, such as C=N/C–N and N–H groups, on its surface, and the enhanced conjugation effect contributed to the increased fluorescence activity of the molecule.

### 3.5 TD-DFT studies

In order to create and formulate the UV-absorbing compounds, an understanding of the electronic transitions of these compounds has been utilized, employing a comparative TD-DFT approach for the synthesized compounds being studied. All compounds show diverse peaks at different wavelengths representing electronic transitions.^47,48^ To enhance theoretical understanding, it is recommended to incorporate solvent effects in TD-DFT calculations using implicit solvation models like the Polarizable Continuum Model (PCM). This model accounts for the solvent's dielectric environment, affecting electronic states and excitation energies, which can be compared with vacuum-phase data and experimental results to gain insights into solvent influence on spectroscopic properties and improve predictive accuracy.

**Fig. 3:**
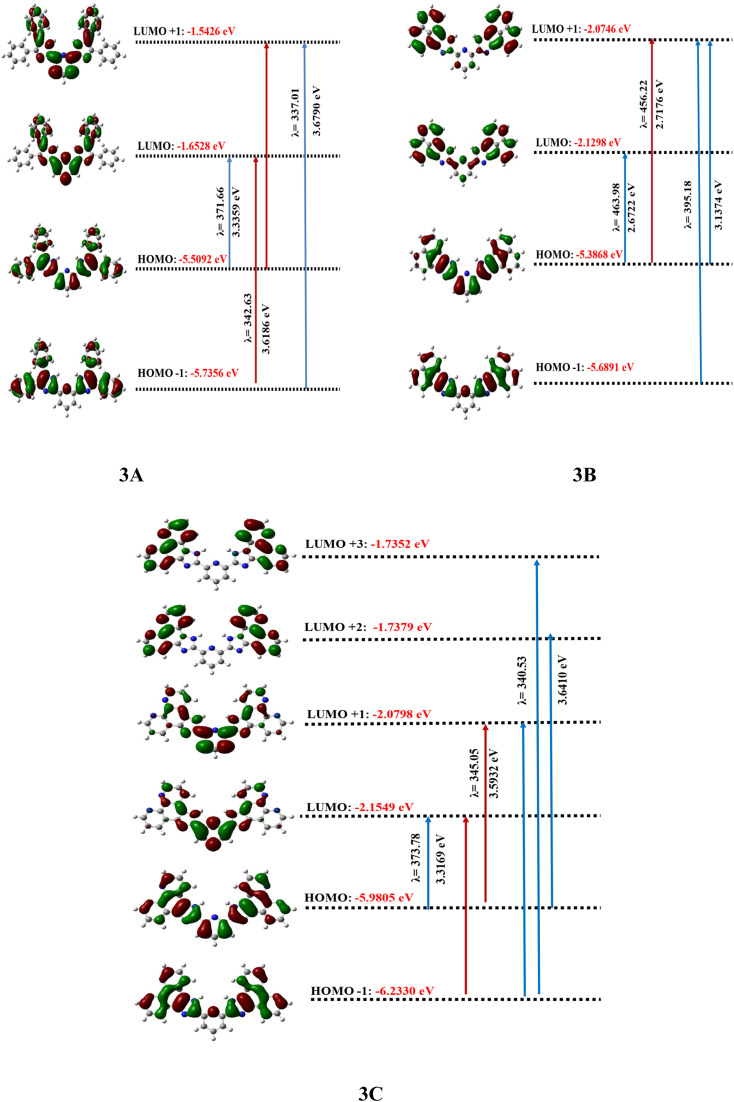
Frontier molecular orbitals involved in the electronic absorption transitions of the compounds 3A–3C calculated at TD-DFT/B3LYP/6-311G(d,p).

This study investigates the structure–activity association by analyzing delocalized π-orbitals and electronic transition energies. Peak absorption wavelengths and their oscillation strengths, which correspond to electronic transitions between frontier molecular orbitals were examined. The HOMO is delocalized across the entire π-conjugated ring system, while the LUMO is primarily located on the pyridine ring. Table 4 summarizes the maximum absorption wavelengths (*λ*_max_), oscillation strengths (*f*), excitation energies (*E*), electronic transition contributions (ETC %), and the deviation between experimental and theoretical values for all compounds. The calculated *λ*_max_ values mostly fall within the range of lower-lying singlet electronic transitions (HOMO → LUMO). Each molecule exhibited three excited states with varying transitions. ETC % values highlight the dominant electronic transition for each excited state shown in Fig. 3. The absorption data were compared to experimental values to confirm the computational outcomes.

**Table 4:** Excitation energies and oscillator strengths of synthesized compound calculated at DFT/B3LYP/6-311G(d,p)

State	Assignment		Coefficient	Energy of transition (eV)	Wavelength (nm)	Oscillator strength
**Excitation energies and oscillator strengths of 3A molecule**						
	From	To				
S_0_–S_1_	HOMO	LUMO	0.70015 (98.04%)	3.3359	371.66	0.3141
S_0_–S_2_	HOMO−1	LUMO	0.50879 (51.77%)	3.6186	342.63	0.0123
	HOMO	LUMO+1	0.48543 (47.12%)	3.6790	337.01	0.2605
S_0_–S_3_	HOMO−1	LUMO+1	0.70062 (98.17%)			

**Excitation energies and oscillator strengths of 3B molecule**						
	From	To				
S_0_–S_1_	HOMO	LUMO	0.63066 (79.54%)	2.6722	463.98	0.0645
S_0_–S_2_	HOMO	LUMO+1	0.59602 (71.04%)	2.7176	456.22	0.0334
S_0_–S_3_	HOMO−1	LUMO	0.60521 (73.25%)	3.1374	395.18	00
	HOMO	LUMO+1	0.35801 (25.63%)			

**Excitation energies and oscillator strengths of 3C molecule**						
	From	To				
S_0_–S_1_	HOMO	LUMO	0.70123 (98.34%)	3.3169	373.78	0.5240
S_0_–S_2_	HOMO−1	LUMO	0.38383 (29.64%)	3.5932	345.05	0.0525
	HOMO	LUMO+1	0.58229 (67.81%)			
S_0_–S_3_	HOMO−1	LUMO+1	0.63417 (80.43%)	3.6410	340.53	0.3492
	HOMO−1	LUMO+3	0.11034 (24.34%)			
	HOMO	LUMO+2	0.25695 (13.20%)			

TD-DFT calculations were performed on molecules 3A, 3B, and 3C. Three bands at 342.63, 371.66, and 337.01 nm, which correspond to HOMO → LUMO, HOMO−1 →LUMO, and HOMO−1 → LUMO+1 in that order, define the theoretical range of 3A. The shift observed at 342.63 nm results from a 98.04% contribution from the HOMO to LUMO transition. In contrast, the subsequent excitation band at 371.66 nm arises from a 51.77% contribution from the HOMO−1 to LUMO transition. Furthermore, the third excitation band at 337.01 nm corresponds to a 98.17% contribution from the HOMO−1 to LUMO+1 transition.^49^ As a result, the sole transition states that have effective oscillator strengths of 0.3141, 0.0123, and 0.2605 are S0 → S1, S0 → S2, and S0 → S3, in that order. Fig. 3 illustrates how the FMO orbitals of compound 3A and the movement of electron density influence the electronic transitions. Compound 3B's TD-DFT Fig. 3 shows three bands at wavelengths of 463.98, 456.22, and 395.18 nm that are caused by the transitions HOMO → LUMO, HOMO → LUMO+1, HOMO−1 → LUMO+1 and HOMO → LUMO+1.

TD-DFT simulations forecasted one strong band and two weaker bands overall. From Table 4, it can be inferred that in the gas phase, bands II and III of all three molecules exhibited low oscillator strength (*f*) values. This suggests that these two bands are of low intensity and involve a forbidden transition. Conversely, band I across all solvents showed moderate oscillator strength values. This implies that band I is more intense compared to bands II and III, indicating it is an allowed transition.

The initial excitation band is observed at 463.98 nm, which is attributed to a 79.54% contribution from the transition HOMO → LUMO. The subsequent excitation band at 456.22 nm shows a 71.04% contribution from the transition HOMO → LUMO+1. The third band, located at 395.18 nm, is associated with contributions of 73.54% from the transition HOMO−1 → LUMO+1 and 25.63% from the HOMO → LUMO+1 transition. The only allowed transition states that exhibit significant oscillator strengths of 0.0645, 0.0334, and 0 were the vertical excitation energy states S0 → S1, S0 → S2, and S0 → S3, respectively. For the theoretical spectrum of compound 3C, the transition occurring at 373.78 nm is attributed to a 98.34% contribution from the HOMO → LUMO transition. In contrast, the second excitation band at 345.05 nm is due to contributions of 29.64% from the HOMO−1 → LUMO transition and 67.81% from the HOMO → LUMO+1 transition. The third excitation band, found at 340.53 nm, corresponds to contributions of 80.43%, 24.34%, and 13.20% from the transitions HOMO−1 → LUMO+1, HOMO−1 → LUMO+3, and HOMO → LUMO+2, respectively. The only valid transition states with relevant oscillator strengths of 0.5240, 0.0525, and 0.3492 correspond to the vertical excitation energy states S_0_ → S_1_, S_0_ → S_2_, and S_0_ → S_3_. In Fig. 3, the orbitals of the frontier molecular orbitals (FMO) and the electron density transfer of compound 3C, which are pertinent to the electronic transitions, are discussed.

### 3.6 Charge-transfer integral

The charge-transfer integral demonstrates the internal arrangement within a molecule and represents the simplicity of charge movement. Elevated values of the charge integral suggest that the pathway for charge mobility contains fewer irregular states. The values of the charge integral were calculated utilizing the subsequent equations.^50,51^
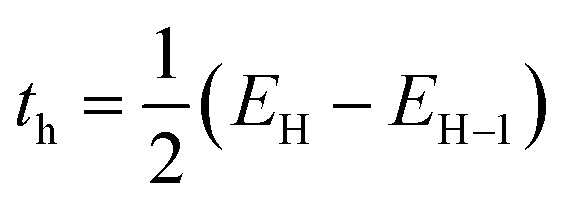

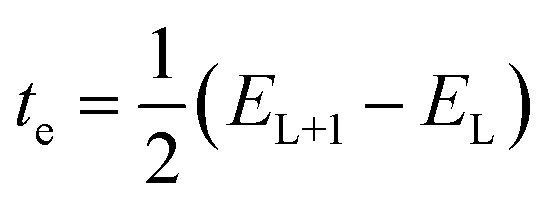


In this context, *E*_H_ and *E*_L_ represent the energy levels of the Highest Occupied Molecular Orbital (HOMO) and Lowest Unoccupied Molecular Orbital (LUMO) measured in electron volts (eV). The term *E*_H−1_ refers to the energy of the orbital that lies one level below the HOMO, while *E*_L+1_ indicates the energy of the orbital that is one level above the LUMO. Additionally, it presents the transfer integral for electrons (*t*_e_) and holes (*t*_h_), highlighting an improved rate of electron mobility for the molecule (shown in SI file Table 7). The table presents the calculated charge-transfer integral values (*t*_e_ and *t*_h_) and the ground state dipole moments for three different molecules, 3A, 3B, and 3C. The electron transfer integral (*t*_e_) for molecule 3A (0.0551) is notably higher than that for 3B (0.0276) and 3C (0.0376), suggesting potentially more efficient electron transport in 3A. Conversely, the hole transfer integral (*t*_h_) is highest for molecule 3B (0.1512), indicating a more facile hole transfer compared to 3A (0.1132) and 3C (0.1263). This suggests that 3B might be a better hole transporter, while 3A could be more suited for electron transport. Regarding the ground state dipole moments, molecule 3A exhibits the largest value (3.7459 Debye), followed by 3B (3.4124 Debye), and 3C has the smallest dipole moment (1.2903 Debye). The significant difference in dipole moments, particularly the much lower value for 3C, could indicate differences in molecular polarity and charge distribution, which impact their interactions with polar environments or their self-assembly properties.

### 3.7 Density of state analysis (DOS)

**Fig. 4:**
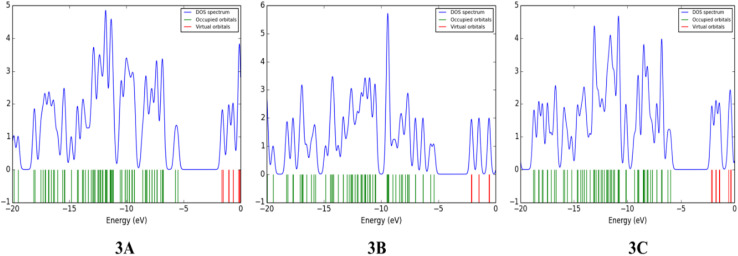
Density of states plot of 3A, 3B and 3C calculated at DFT/B3LYP/6-311G(d,p).

The DOS analysis in quantum mechanics elucidates the presence of energy states for ongoing transitions, happening in increments of energy levels per unit. The DOS graphs illustrate the entire energy positions represented as a range beneath the spectral peaks. The density of states (DOS) spectrum for the optimized compounds was obtained using the Gauss Sum 3.0 program^52^ and is shown in Fig. 4.

This spectrum visually represents electron behavior within the conduction and valence bands, revealing the distribution of energy states. The segments at the beginning of the energy axis of the graph, ranging from −20 eV to −5 eV, are referred to as filled orbitals, while the range from −5 eV to 0 eV is known as virtual orbitals. Virtual orbitals are unoccupied and are often termed acceptor orbitals. On the other hand, filled orbitals are identified as donor orbitals. A pronounced density of states (DOS) at certain energy points indicates a high availability of states for occupation. Conversely, a DOS of zero intensity signifies that no states are available for the system to occupy. The overarching blue curve, the DOS spectrum, indicates the density of states at each energy level. A complete analysis would involve discussing the orbital density distribution for key orbitals, particularly the HOMO and LUMO. For instance, if the HOMO is localized on a specific part of the molecule and the LUMO on another, it signifies a charge-transfer character. Such a spatial separation between donor and acceptor regions, visible from the orbital density, is directly linked to the efficiency of intramolecular charge transfer. This efficiency, in turn, impacts the molecule's fluorescence behavior^53^ DOS diagrams (Fig. 4) display molecular orbital energy values on the *x*-axis and the relative strength of states on the *y*-axis. These diagrams for compounds 3A–3C indicate that the HOMO and LUMO are primarily derived from the donor portion, with a minor contribution from the acceptor. The band gap values shown are consistent with those calculated and shown in the SI file (Table 8). A notable overlap between the donor-localized highest occupied molecular orbital and the acceptor-localized lowest unoccupied molecular orbital, combined with a suitable energy difference, promotes effective electron transfer when excited, which has a direct effect on phenomena such as fluorescence. A pronounced charge-transfer characteristic in the excited state, which can be recognized by specific orbital distribution on the donor and acceptor fragments in the density of states (DOS)-projected molecular orbitals, typically results in a diminished oscillator strength and, as a result, weaker or redshifted fluorescence, since the emission involves a transition from a significantly charge-separated state. In contrast, if the excited states maintain considerable local excitation characteristics (less charge separation), one might observe more intense fluorescence.

### 3.8 UV-visible studies

**Fig. 5:**
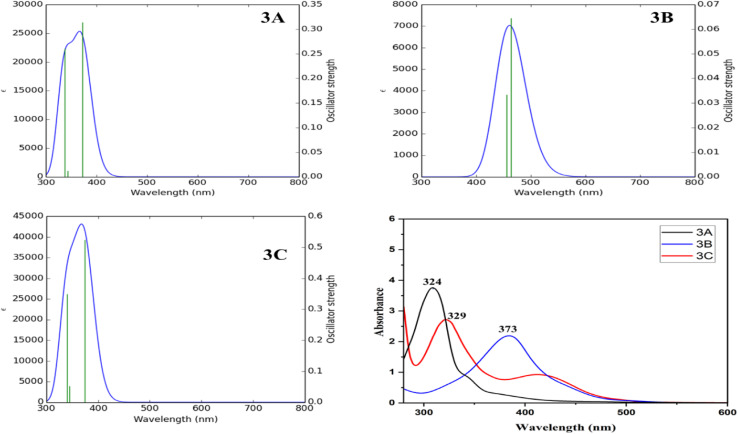
The experimental UV-vis spectra and theoretical spectra calculated at DFT/B3LYP/6-311G(d,p).

The absorption spectra of the studied imidazole derivatives were measured in ethanol. In general, the samples dissolved well in ethanol and typically exhibited wide absorption bands in the UV-visible spectrum. The theoretical and experimental graphs are shown in Fig. 5. The theoretical absorption spectrum of 3A, 3B and 3C compounds exhibited a pronounced peak at 371.66, 463.98 and 373.78 nm, while the experimental absorption spectrum was observed at 324, 373 and 329 nm, respectively. Upon switching to different derivatives, nearly identical spectral profiles were noted with only a slight variation in shift. The findings are fairly comparable to the experimental data, revealing errors of 12.66%, 19.43%, and 11.79%, respectively, when comparing theoretical and experimental values, as shown in the SI file (Table 8).

### 3.9 Fluorescence analysis

Iron is a crucial element that has a vital function in biological systems, including its roles in oxygen absorption, oxygen utilization and the process of electron shifting serving as an accessory component in numerous enzymatic processes. Nevertheless, an imbalance in iron levels within living organisms is linked to serious health issues such as anemia, heart failure, cellular injury, liver impairment, and more. Consequently, the identification of Fe is essential for tracking well-being concerns and diagnosing illnesses. Although Fe can be found in both Fe^2+^ and Fe^3+^ states, Fe^3+^ is generally more stable and has been extensively researched as an analyte in fluorescent sensors.^54^

#### 3.9.1 pH detection

**Fig. 6:**
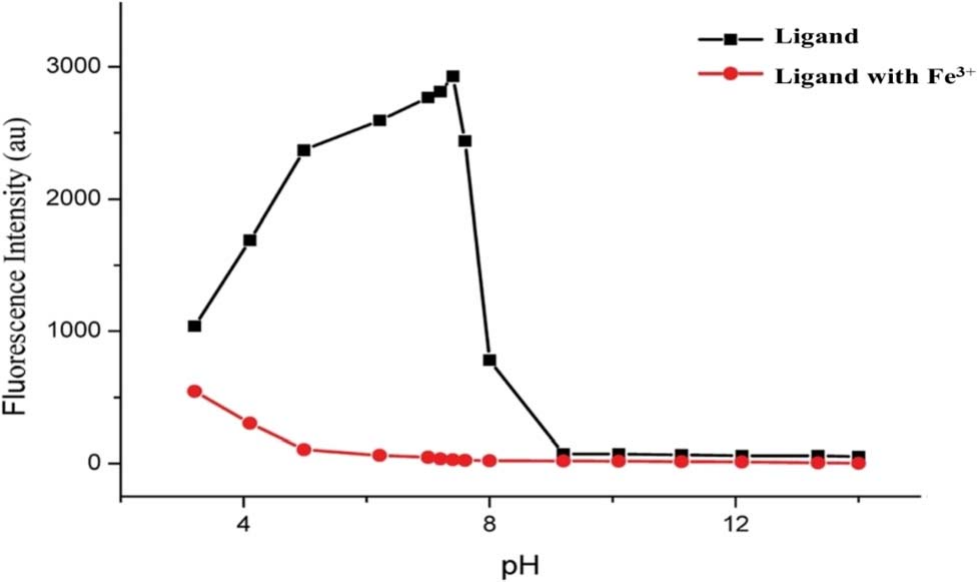
pH effect on the fluorescence intensity at 350 nm of 3A ligand (100 ppm) and ligand (100 ppm) with Fe^3+^ (100 ppm) in ethanol.

The variation of fluorescence intensity of the sensor of ligand 3A taken as a reference with pH in the appearance and nonappearance of Fe^3+^ ions is provided in Fig. 6. The pH level was modified using 1 N HCl or 1 N NaOH solution. At a pH of 3 to 8, the ligand exhibited intense fluorescence, whereas the combination of ligand and Fe^3+^ displayed only faint fluorescence. In contrast, at pH levels above 8, the ligand demonstrated reduced fluorescence. Therefore, the optimal pH range for detecting Fe^3+^ was determined to be between 4 and 8, during which the free ligand maintained consistent and strong fluorescence, and the fluorescence quenching was at its lowest with the introduction of Fe^3+^.

#### 3.9.2 UV-vis spectral response of the sensor to Fe^3+^

**Fig. 7:**
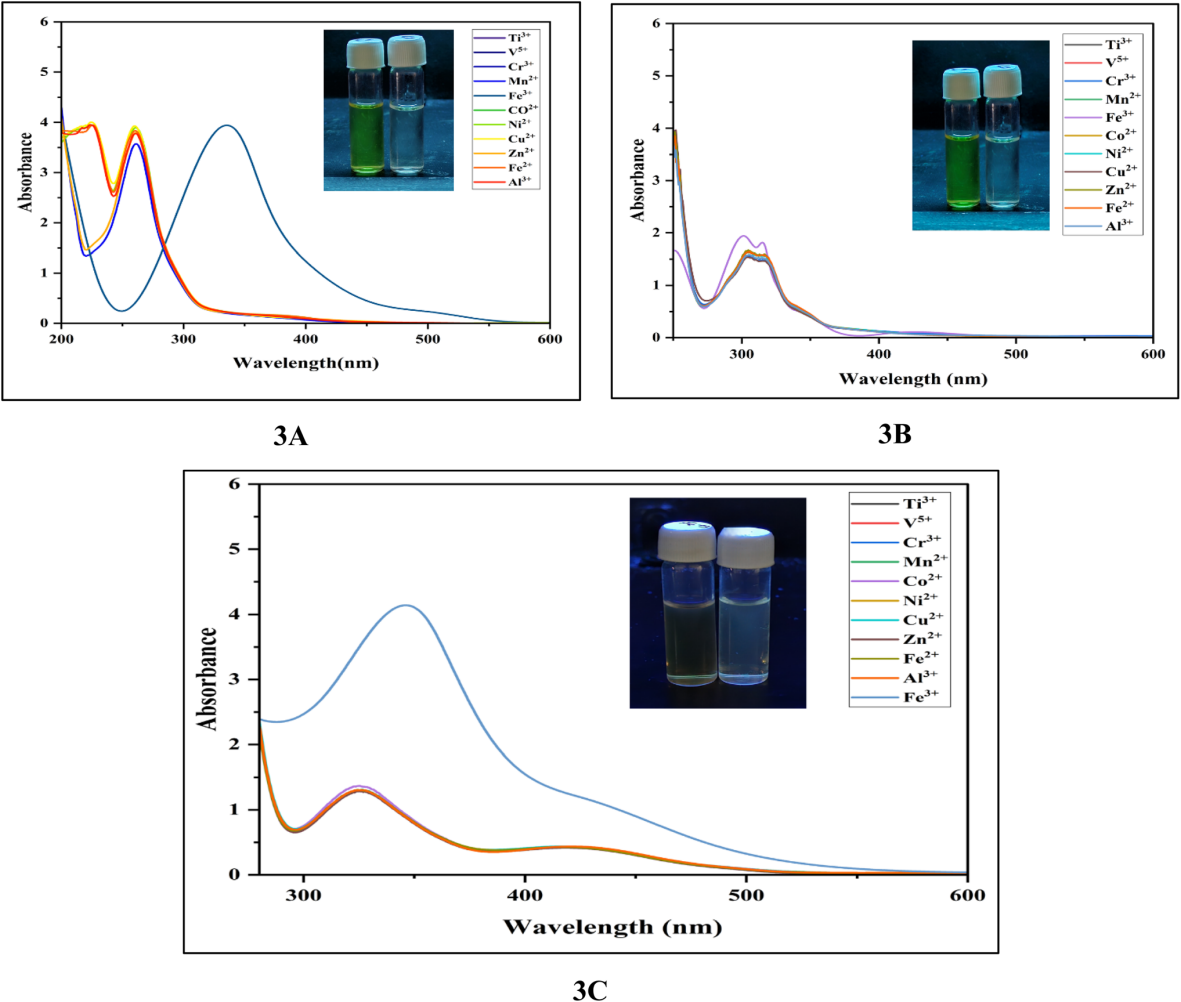
UV-vis spectra of 3A, 3B and 3C with the addition of different metal ions (100 ppm) in ethanol at pH 7.5. Inset: colour change of the probe in the absence and presence of Fe^3+^ ions.

The exceptional selectivity and sensitivity of ligands 3A–3C toward active metal ions were validated through optical analysis (UV-vis), as illustrated in Fig. 7. Ligands 3A–3C did not exhibit notable alterations upon the introduction of different metal ions such as Ti^3+^, V^5+^, Cr^3+^, Mn^2+^, Co^2+^, Ni^2+^, Cu^2+^, Zn^2+^, Fe^2+^ and Al^3+^. In contrast, significant changes in their absorption spectra were observed when Fe^3+^ ions were present in an ethanol medium at a pH of 7.5.

All absorption spectra showed no notable changes, except for the addition of Fe^3+^, which caused a blue shift from 290 nm to 385 nm and a colour transition from colourless to yellow. Initially, when we introduced 2.0 equivalents of Fe^3+^ ions to a bare receptor 3A, a new complex absorption peak at 345 nm was detected. Similarly, we assessed the selectivity and sensitivity of 3B and 3C towards the aforementioned active metal ions. Both ligands showed considerable responses to Fe^3+^ ions, with complex absorption peaks observed at 320 nm and 353 nm, while no notable changes were seen in the presence of other tested competitive ions. Clear isosbestic points centered at 345 nm for the 3A ligand, 320 nm, and 353 nm for 3B and 3C respectively, indicate that the metal complexes formation as shown in Fig. 7. All three ligands possessed appropriate requisite sites like amine, nitrogen from the pyridine ring, and a cavity that allows for metal ion complexation, controlling ion selectivity.^55,56^

#### 3.9.3 Fluorescence responses of sensors to Fe^3+^

**Fig. 8:**
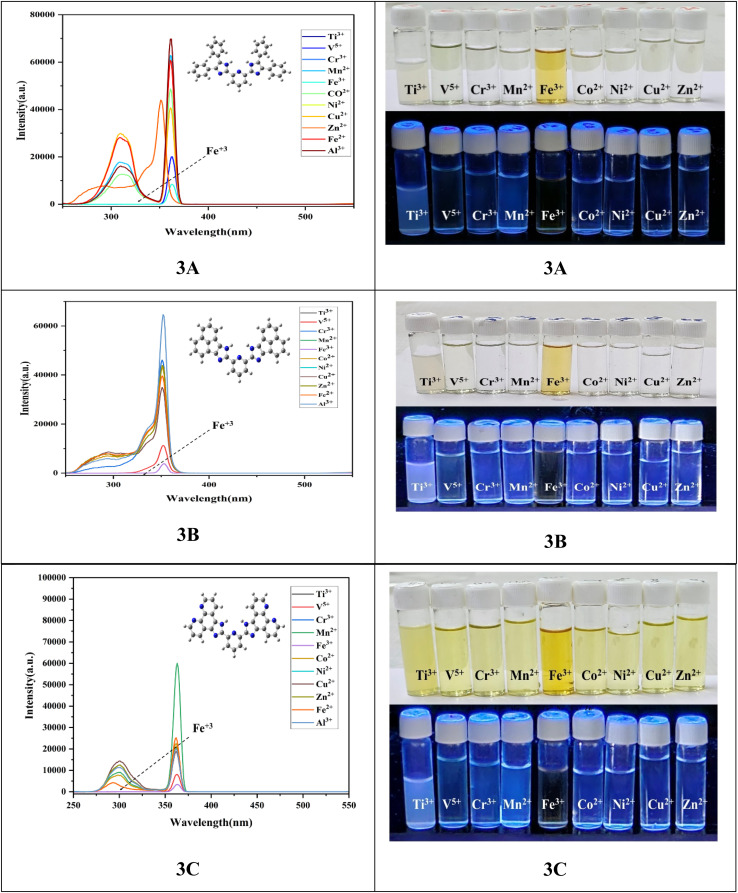
Fluorescence spectra of all sensor 3A–3C with the adding of diverse metal ions in ethanol (pH-7.5) and the ligands with different metal ions (100 ppm), under UV light.

The sensing ability of the sensor of all three ligands was explored by fluorescence trials in the presence of numerous metal ions (100 ppm), together with Ti^3+^, V^5+^, Cr^3+^, Mn^2+^, Fe^3+^, Co^2+^, Ni^2+^, Cu^2+^, Zn^2+^, Fe^2+^ and Al^3+^ in ethanol at pH 7.5, as shown in Fig. 8. Moreover, the probes have been used to detect Fe^+2^ and Al^+3^. The result shows that there is no selectivity and sensitivity of these ions towards the ligands.

The fluorescence spectra were recorded between 250 nm and 350 nm (*λ*_ex_: 300 nm). Each of the three sensors exhibited significant fluorescence quenching in the presence of Fe^3+^ when contrasted with other metal ions, leading to a distinct colour alteration from colourless to vibrant yellow. These results indicate that the ligand sensor exhibits a significant sensitivity and specificity for identifying Fe^3+^ ions, accompanied by a noticeable colour change that is likely beneficial for detection by the unaided eye. The probe's emission was determined to arise from the enhanced conjugation within the ring, which features the highest π-conjugation and the lowest HOMO–LUMO energy gap. Since molecules with delocalized electronic clouds can readily coordinate with metal ion systems, the hierarchy should be 3C > 3B > 3A. Ultimately, we can conclude that the highest level of conjugation in the ligand is directly related to the probe's emission. The emission intensity of the probes in an ethanol solution increased following the introduction of the metal cation, and the probe solution changed to a vibrant yellow due to the formation of a ligand to metal charge-transfer complex with Fe^3+^. It also demonstrated a strong selectivity for Fe^3+^ and an impressive fluorescence “turn-on” response.

#### 3.9.4 The possible mechanism of the sensors with Fe^3+^

**Scheme 2:**
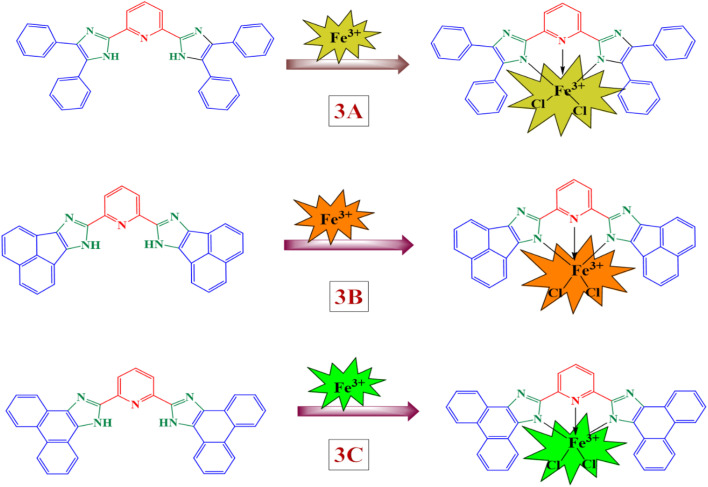
The possible mechanism of sensors with Fe^3+^ ions.

As mentioned earlier, the ligand demonstrated outstanding selectivity for Fe^3+^. The alteration in colour noted for the probe containing Fe^3+^ is linked to the ring-opening mechanism depicted in Scheme 2. It is reasonable to suggest that Fe^3+^ may engage with the nitrogen atom in the pyridine ring as well as the π-orbital of 1*H*-imidazole present in the ligand. Notably, there is a significant variation in the binding with the sensors, as conjugation increases in the order of 3C > 3B > 3A, which exhibited the highest emission enhancement in comparison to Fe^3+^. This specifies that the nitrogen in the imidazole ring is crucial for the metal–ligand interaction with Fe^3+^.^57,58^ In addition, the binding interaction ratio between the ligand and Fe^3+^ ions was evaluated using Job's plot.

Once the selectivity of the ligand has been established, it is crucial to examine the coordination mode between the ligand and Fe^3+^ to better understand how the ligand recognizes Fe^3+^. Job's method was utilized to analyze the binding stoichiometry between the ligand and Fe^3+^. Solutions containing varying ratios of the ligand and Fe^3+^ were prepared. The emission spectrum for each solution was recorded, and from the resulting emission spectra, a Job's plot was generated. The Job plots for all three ligands obtained through fluorescence titrations demonstrated a peak emission intensity at approximately 0.5 mol fractions, suggesting that the sensor forms a 1 : 1 complex with Fe^3+^.^59–61^ (provided in the SI file Spectrum 17–19).

#### 3.9.5 DFT analysis of proposed ferric complex

**Fig. 9:**
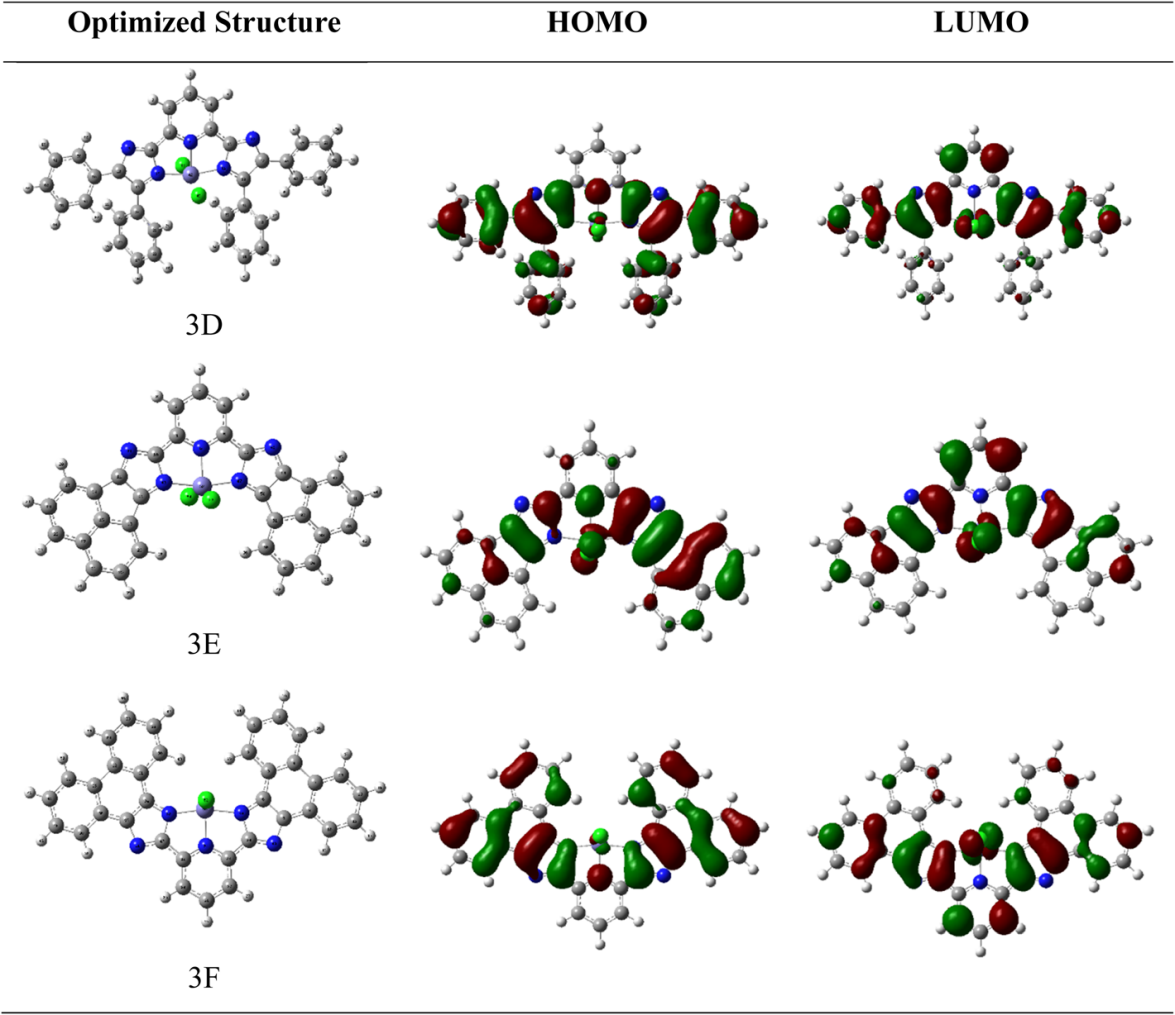
Optimized geometries and FMO orbital of studied proposed ferric-based complexes at the DFT/B3LYP/LanL2DZ level of theory.

DFT calculations were performed to investigate this proposed mechanism at the unrestricted B3LYP level of theory, in combination with a LanL2DZ basis set suitable for single-point energy calculations of the complex. Optimizations were performed in the gas phase. The optimized structures and FMO of ferric-based complexes are given in Fig. 9.

In complexes 3D, 3E, and 3F specifically, there are three Fe–N bonds and two Fe–Cl bonds. The measured bond lengths for complex 3D fall between 1.8749 Å and 2.3072 Å. Similarly, complex 3E exhibited bond lengths ranging from 1.8762 Å to 2.2795 Å. Furthermore, the bond lengths in complex 3F were found to range from 1.8734 Å to 2.2866 Å. The HOMO energies for the complexes examined range from −5.6716 eV to −5.8346 eV, whereas the LUMO energies fall between −4.9412 eV and −5.0425 eV. The observed energy gaps (Δ*E*) for complexes 3D, 3E, and 3F are 0.7303, 0.8147, and 0.7921 eV, respectively, with complex 3D having the smallest energy gap as shown in Table 5.

**Table 5:** Global reactivity parameter of metal–ligand complex calculated at DFT/B3LYP/LanL2DZ basis set

Molecules	3D	3E	3F
Optimized energy (Hartree)	−1774.4996	−1617.2582	−1772.1424
Dipole moment (D)	1.2338	0.6349	0.9235
HOMO (eV)	−5.6716	−5.7570	−5.8346
LUMO (eV)	−4.9412	−4.9423	−5.0425
Band gap (eV)	0.7303	0.8147	0.7921
Ionization energy (eV)	5.6716	5.7570	5.8346
Electron affinity (eV)	4.9412	4.9423	5.0425
Chemical hardness (eV)	0.3652	0.4073	0.3961
Global softness (eV)	1.3692	1.2274	1.2624
Chemical potential (eV)	−5.3064	−5.3497	−5.4385
Electronegativity (eV)	5.3064	5.3497	5.4385

For complexes 3D and 3F, the HOMO cloud density is uniformly spread across the molecule, while in complex 3E, the HOMO cloud is predominantly concentrated on the imidazole. On the other hand, the LUMO cloud density across all examined complexes is located on the imidazole rings, suggesting the electroactive or reactive properties of the ring.

**Fig. 10:**
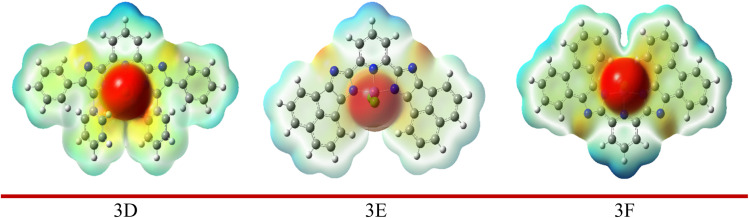
MEP metal–ligand complex calculated at DFT/B3LYP/LanL2DZ basis set.

The transition that occurs from the HOMO to the LUMO is associated with the π–π* transitions of the imidazole ring and Fe^3+^ ions, respectively. However, in complex 3E, the HOMO is mainly situated on the imidazole ring, whereas the LUMO is present on both the Fe^3+^ ion and the imidazole ring, suggesting an n–π* transition. The overall charge transfer in all the complexes analyzed moves from the ligand to the metal. The MEP of all three complexes was illustrated in Fig. 10; the prominent red sphere at the center of the image signifies a zone of high electron density. This area is rich in electrons and is likely linked to the central metal atom and the ligands that are directly attached to it. This zone would be the most appealing to positively charged entities, which could be regarded as the most nucleophilic section of the molecule. The blue regions, especially at the upper part of the molecule near the hydrogen atoms, denote an area of low electron density. These regions are deficient in electrons and are likely where a positive charge is concentrated. This section of the molecule would be the most attractive to negatively charged entities, which could be viewed as the most electrophilic section of the molecule.

## 4 Conclusion

The current research detailed the innovative synthesis of an imidazole derivative acting as chemosensors (3A–3C). The identification of the synthesized products was validated through FTIR, ^1^H NMR, ^13^C NMR spectroscopy and mass spectrometry. The molecular configurations of ligands 3A–3C were optimized utilizing the DFT/B3LYP/6-311G(d,p) basis set on Gaussian09 software. The calculated optimized geometrical parameters were then compared to a structurally similar compound that exhibits a conformation. Comprehensive analyses of the vibrational spectra were performed, and the mean absolute deviation was applied for error analysis. The observed and calculated frequencies are found to be in good agreement. These compounds exhibit significant conjugation characteristics and a narrow HOMO–LUMO energy gap, indicating they are softer molecules. FMO analysis was used to calculate global reactivity parameters (GRPs), revealing the compound's charge transfer, stability, and reactivity. The MEP shows the nucleophilic and electrophilic sites in the molecule. The relevant electronic transitions were explored using the employing time-dependent density functional calculations. The data represented in the Density of States (DOS) aligns well with the insights drawn from FMO diagrams, suggesting a lesser band gap correlating with higher reactivity, reduced stability, and a softer nature of the molecules. Moreover, DFT investigation was also performed on the studied ferric complexes to correlate their geometric parameters with the experimental outcomes, and a strong agreement was observed. For electronic characteristics, FMO analysis was performed, which reveals the LMCT in the studied ferric complexes.

The synthesized three ligands were used for the detection of the Fe^3+^ metal ions. The pH assessment revealed that the optimal range lies between 4 and 8, suggesting that the sensors can function effectively in both acidic and neutral environments. The absorption spectra exhibited no significant alterations upon introducing 10 equivalents of numerous metal ions, except that the addition of Fe^3+^ caused a blue shift from 250 nm to 350 nm, accompanied by a colour transition from colourless to vibrant yellow. The fluorescence spectra demonstrated a clear quenching effect in response to Fe^3+^. Job plots indicated that the sensors establish a 1 : 1 binding ratio with Fe^3+^. Furthermore, these sensors can facilitate visual detection of Fe^3+^, significantly enhancing their potential applications.

## Author contributions

Rohini R. Suradkar: conceptualization, investigation, methodology, validation, writing – original draft. Dnyaneshwar P. Gholap: writing – review & editing. Aarti V. Belambe: visualization. Machhindra K. Lande: conceptualization, methodology, supervision, validation, writing – review & editing.

## Conflicts of interest

The authors declared that there is no conflict of interest in the publication of this research paper.

## Supplementary Material

RA-015-D5RA04242A-s001

## Data Availability

Data will be made available on request. Supplementary information is available. See DOI: https://doi.org/10.1039/d5ra04242a.
