# Supplementary material for: Synthesis and computational evaluation of imidazole-based functional materials for applications in sensing and detection: modulating electronic effects for enhanced performance
Source: RSC Adv. 2025 Sep 29;15(43):35790–806. doi: 10.1039/d5ra04242a (PMC12477525; doi:10.1039/d5ra04242a)
Supplement: RA-015-D5RA04242A-s001 [file RA-015-D5RA04242A-s001.pdf]

Supplementary Information (SI) for RSC Advances.
This journal is © The Royal Society of Chemistry 2025

Synthesis and Computational Evaluation of Imidazole-Based Functional Materials for Applications in Sensing and Detection: Modulating Electronic Effects for Enhanced Performance

Rohini R. Suradkar, Dnyaneshwar P. Gholap, Aarti V. Belambe and Machhindra. K. Lande*,

Department of Chemistry, Dr. Babasaheb Ambedkar Marathwada University, Chhatrapati Sambhajinagar, Maharashtra, India.

*Corresponding Author Email Id: mkl_chem@yahoo.com

Supporting Information

Content

1: ^{1}H, ^{13}C NMR, FTIR, Mass spectra of the compounds.

2: DFT Study of the compounds.

1. ^{1}H, ^{13}C NMR, FTIR, Mass spectra of the 2,6-bis(4,5-diphenyl-1-imidazole-2-yl) pyridine (3A) Compound

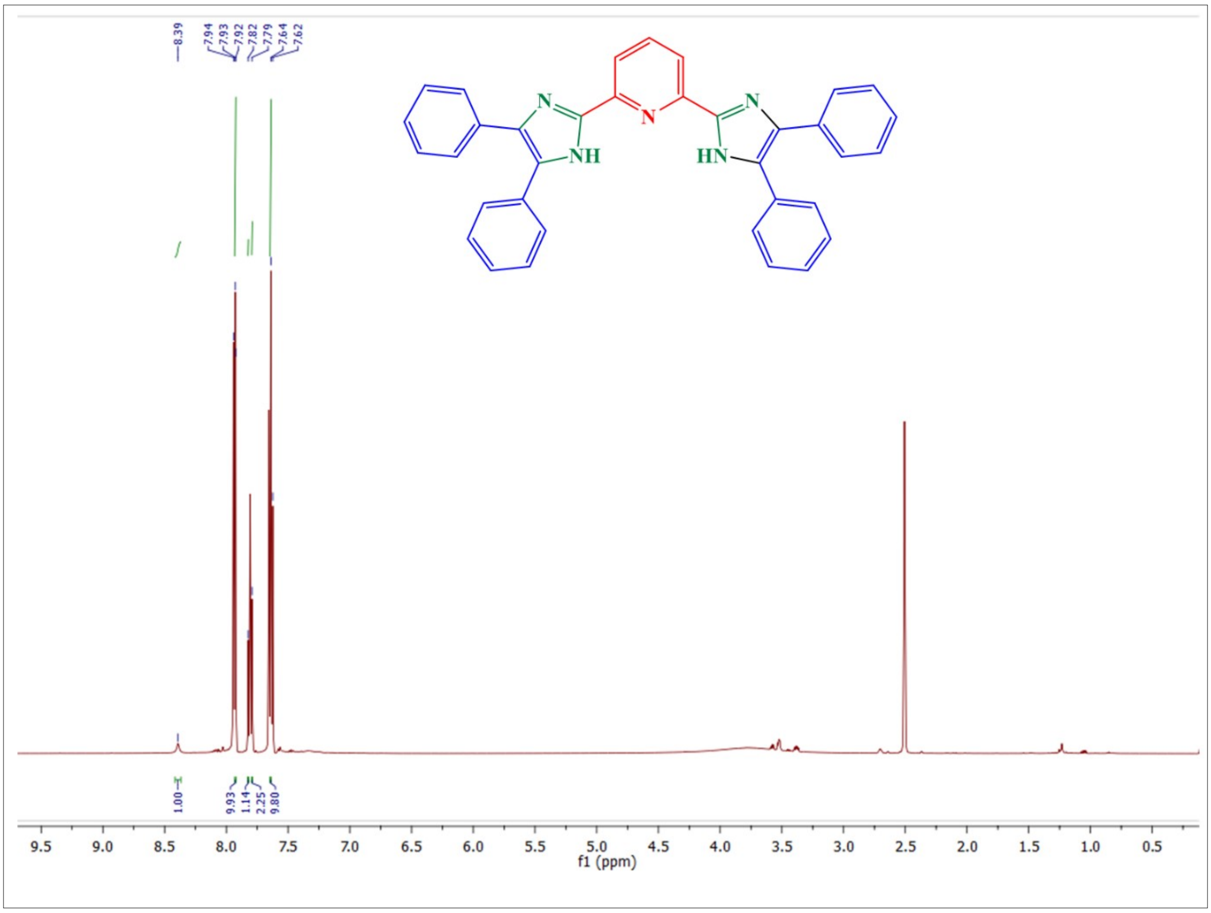

Spectrum 1: ^{1}H NMR Spectrum of 2,6-bis(4,5-diphenyl-1-imidazole-2-yl) pyridine (3A)

^{1}H NMR (500 MH$_{Z}$, DMSO δ ppm): 8.35 (s, 1H), 7.93-7.92 (m, 10H), 7.82 (t, J = 8.5Hz, 1H), 7.79 (d, J = 8.2Hz, 2H), 7.64-7.62 (m, 10H).

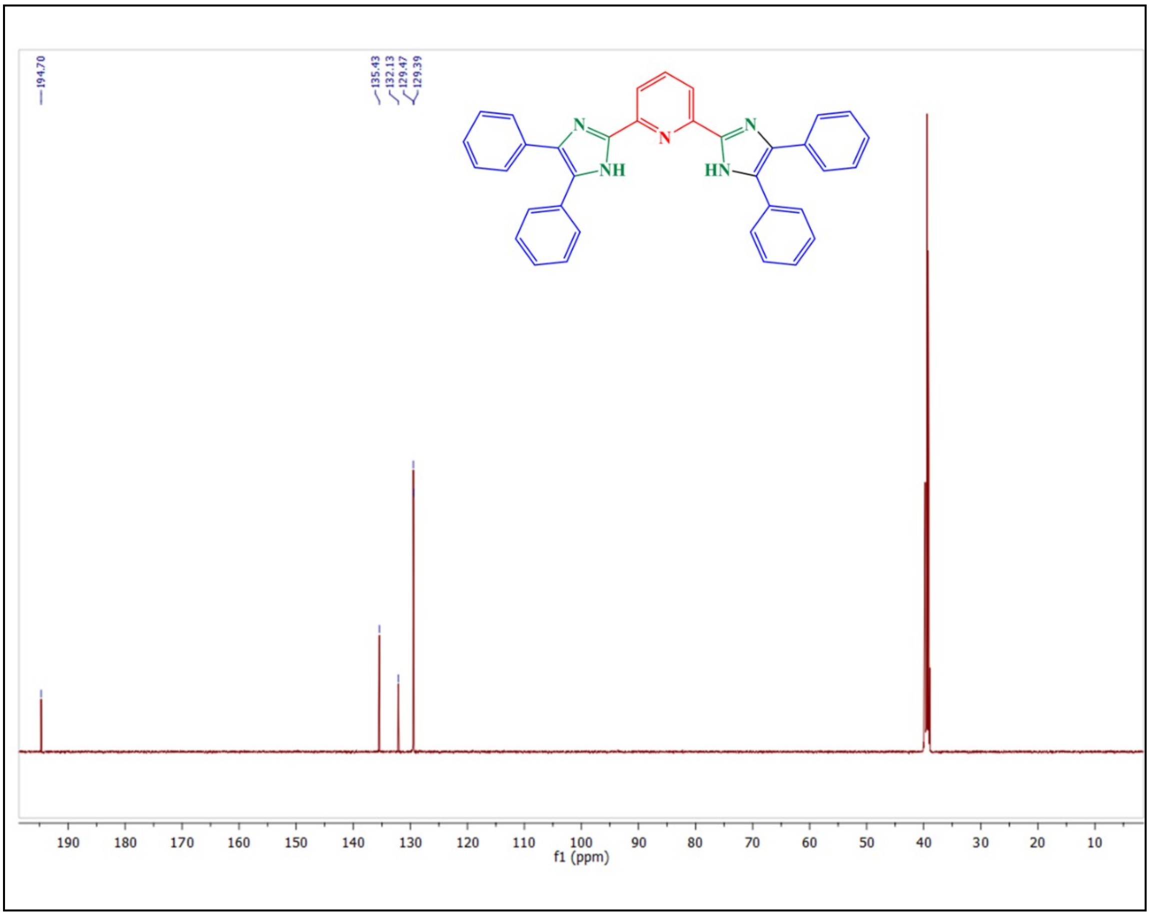

Spectrum 2: ^{13}C NMR Spectrum of 2,6-bis(4,5-diphenyl-1-imidazole-2-yl) pyridine (3A)

^{13}C NMR (500 MHz, DMSO δ ppm): 194.7, 135.4, 132.1, 129.5, 129.4.

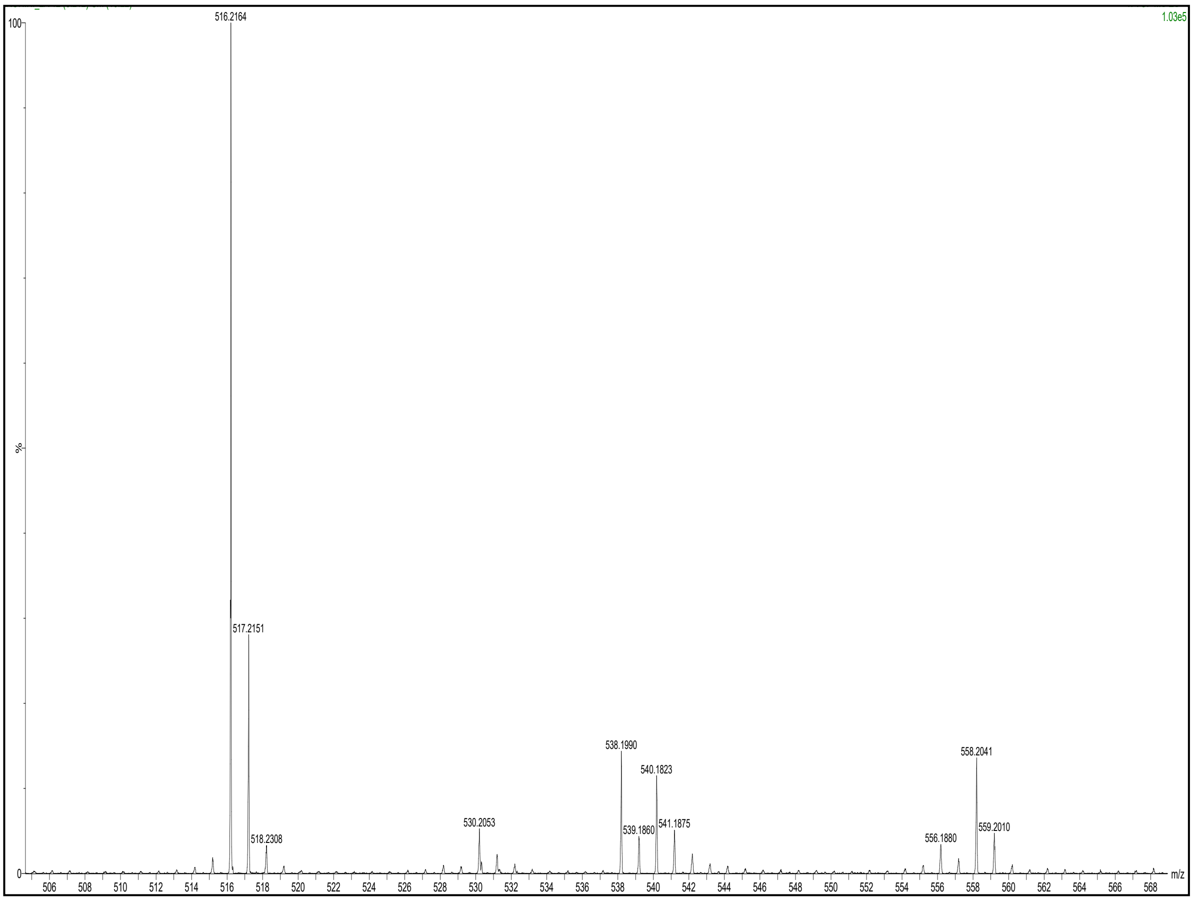

Spectrum 3: Mass spectrum of 2,6-bis(4,5-diphenyl-1-imidazole-2-yl) pyridine (3A)

Mass $C_{33}H_{20}N_8O$, M^+: 516.21.

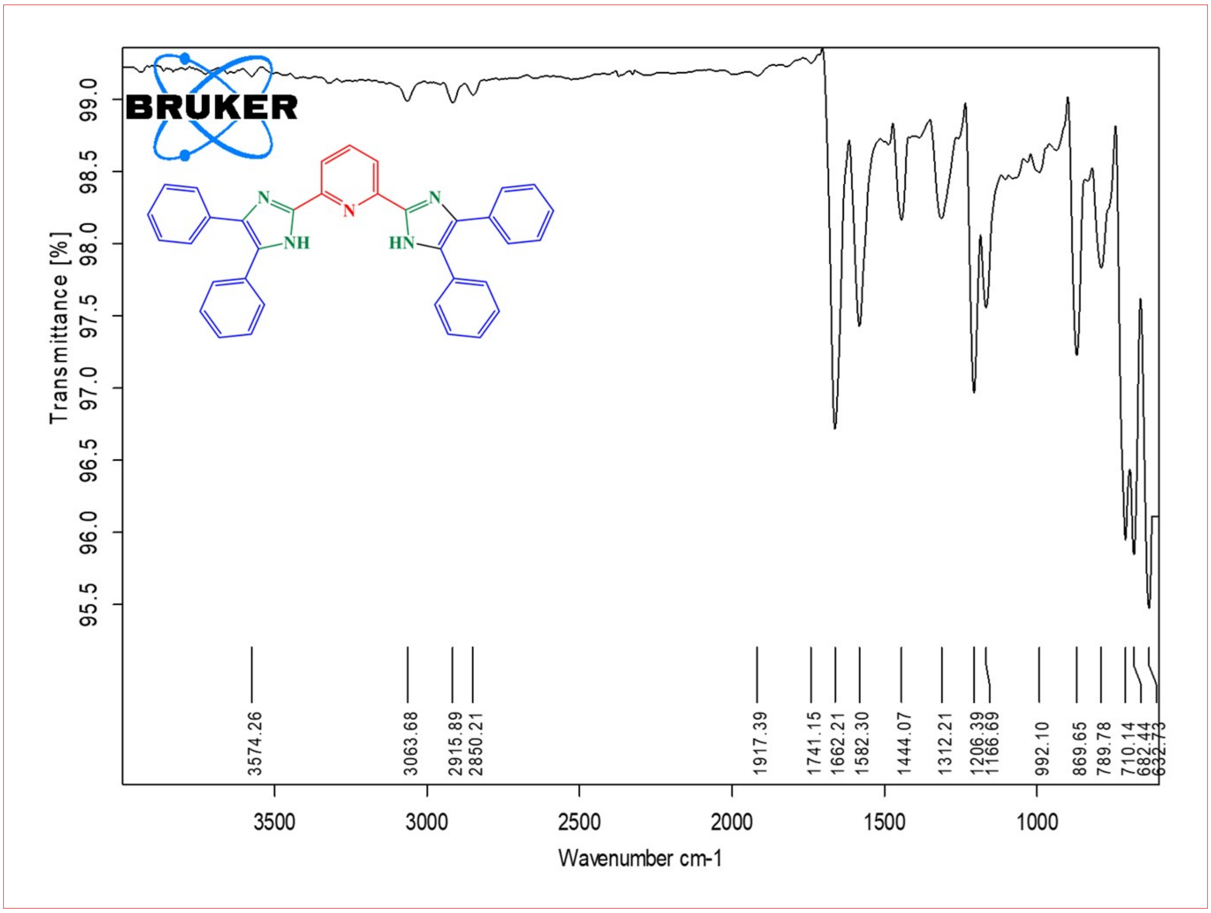

Spectrum 4: FTIR spectrum of **2,6-bis(4,5-diphenyl-1-imidazole-2-yl) pyridine (3A)**

FTIR (υ_{max} cm^{-1}): 3574.26(N-H), 1422.98(C-N), 1582.88 (C=N)

2. ^{1}H, ^{13}C NMR, FTIR, Mass spectra of the 2,6-bis(7H-acenaphtho[1,2-d]imidazol-8-yl)pyridine (3B)

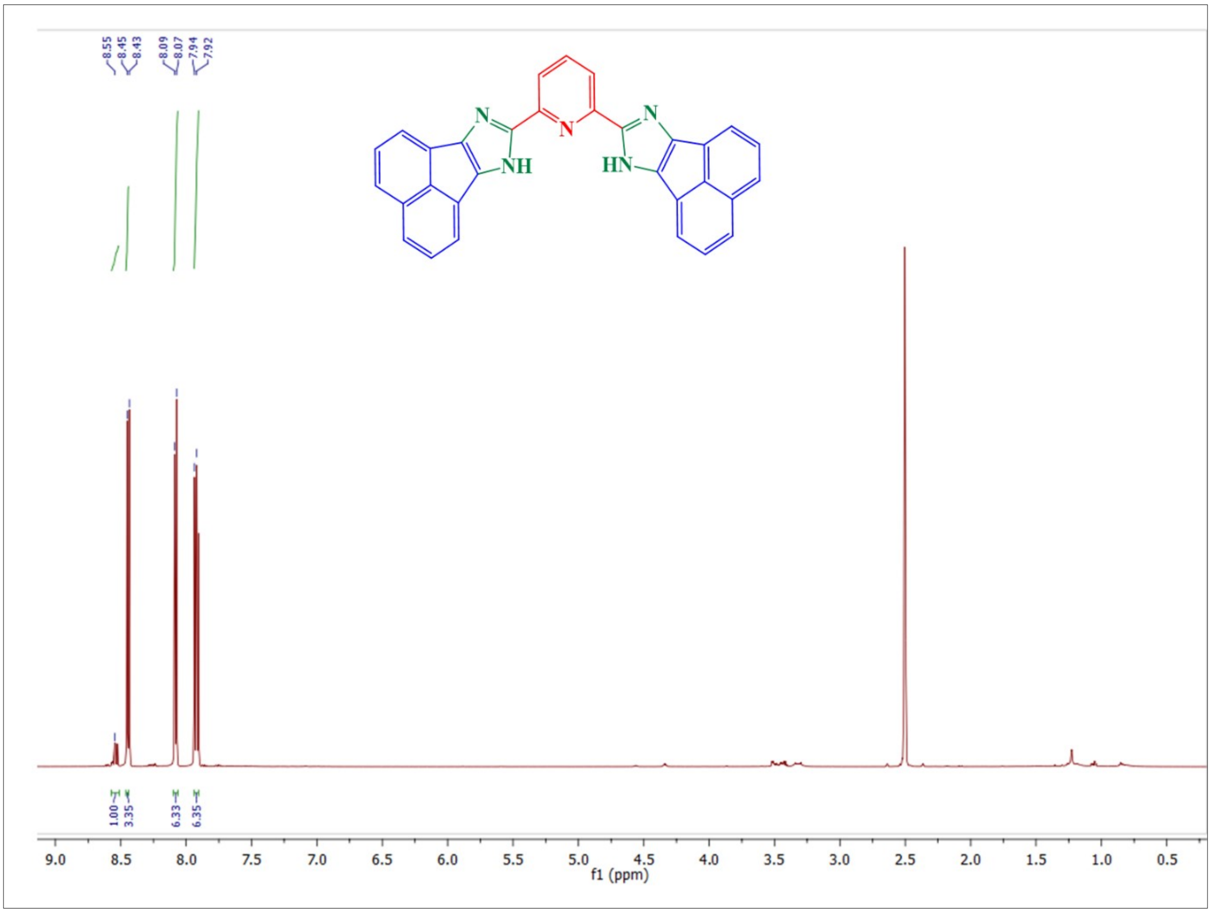

Spectrum 5: ^{1}H NMR spectrum of 2,6-bis(7H-acenaphtho[1,2-d]imidazol-8-yl)pyridine (3B)

^{1}H NMR (500 MH$_{Z}$, DMSO δ ppm): 8.55 (s, 1H), 8.45-8.43 (m, 3H), 8.09-8.07 (m, 6H), 7.94-7.92 (m, 6H).

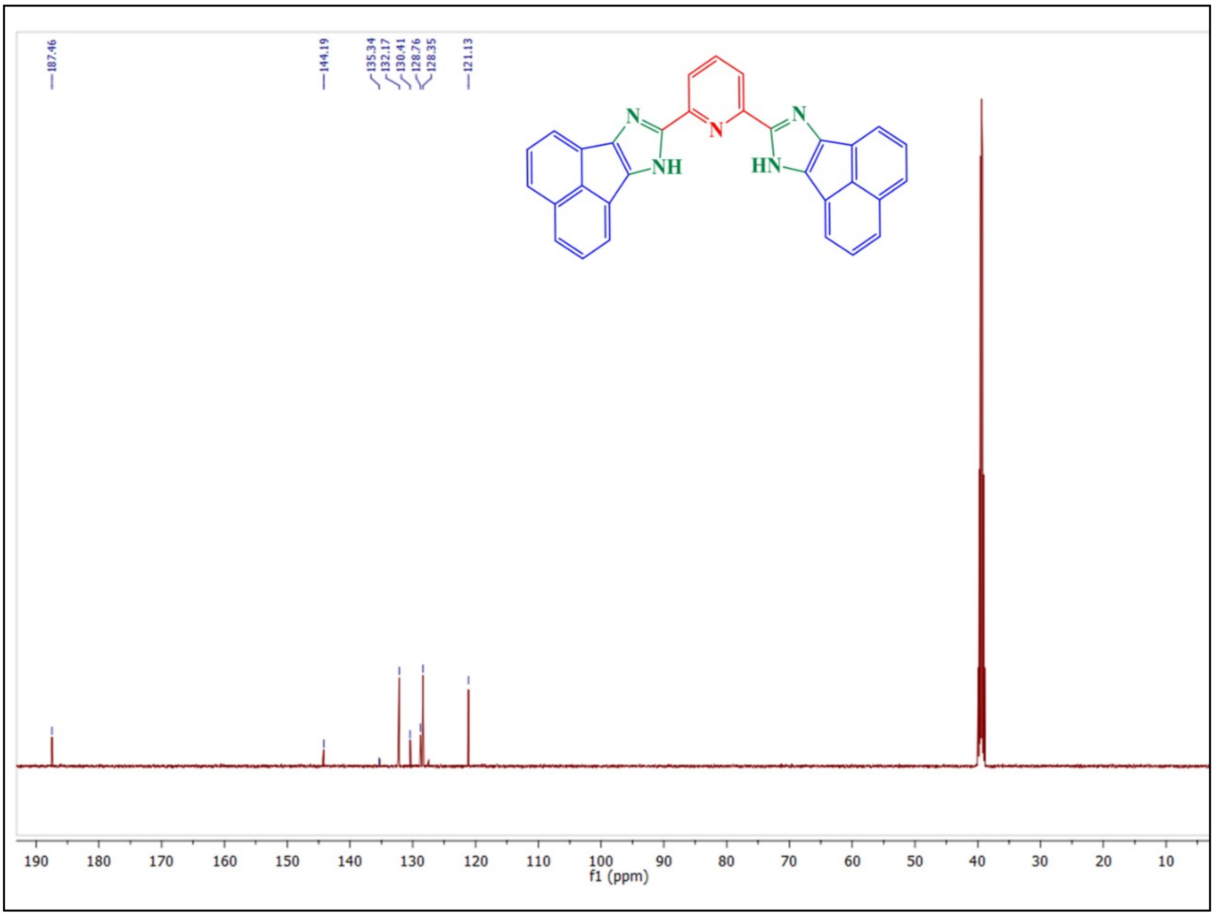

Spectrum 6: ^{13}C NMR spectrum of 2,6-bis(7H-acenaphtho[1,2-d]imidazol-8-yl)pyridine (3B)

^{13}C NMR (500 MHz, DMSO δ ppm): 187.5,144.2, 135.5, 132.2, 130.4, 128.8, 128.4, 121.1.

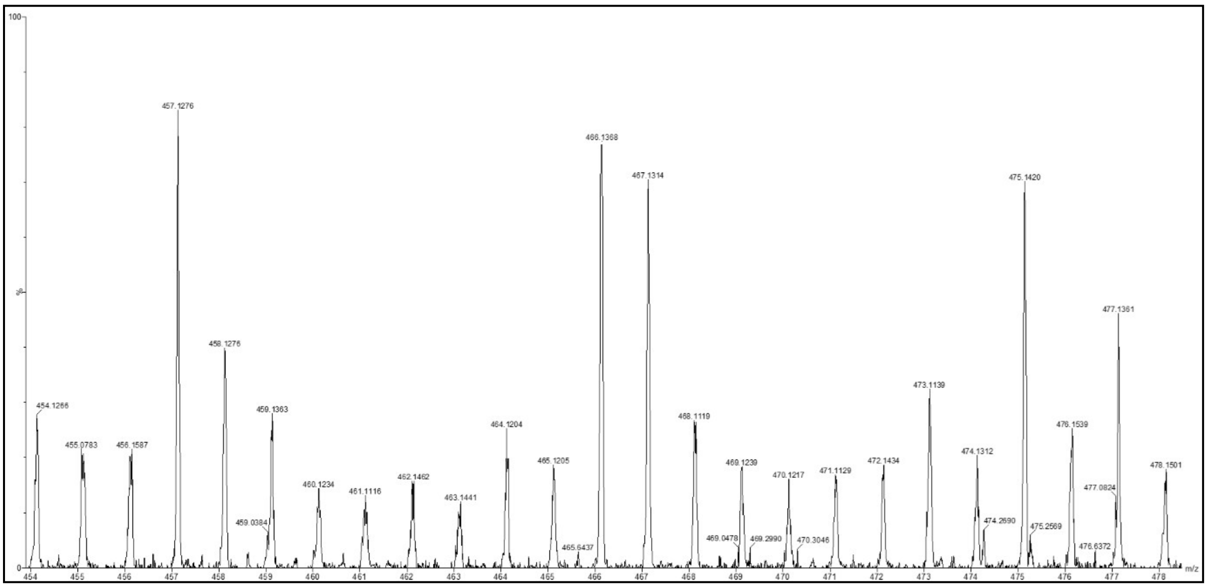

Spectrum 7: Mass spectrum of 2,6-bis(7H-acenaphtho[1,2-d]imidazol-8-yl)pyridine (3B)

Mass $C_{33}H_{20}N_8O$, M^+: 457.12.

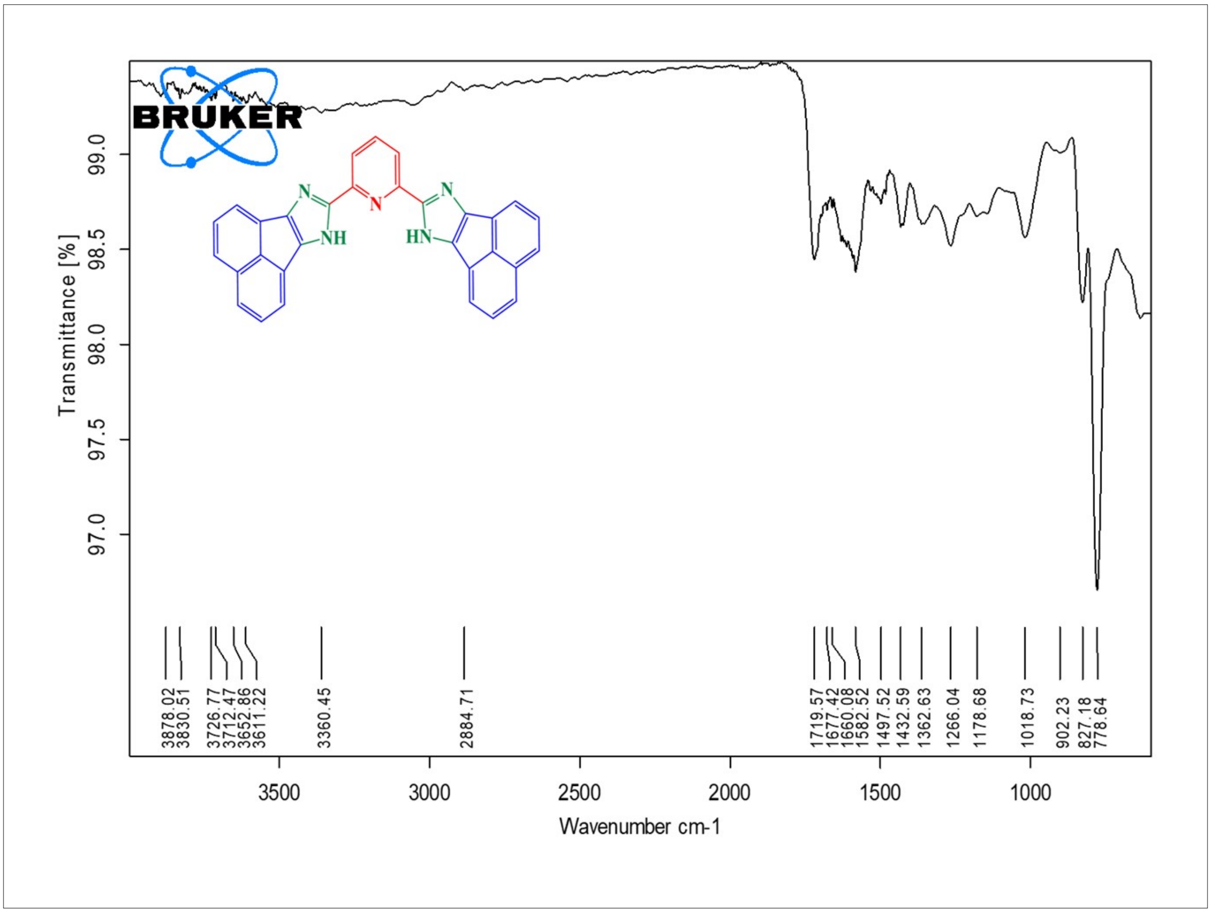

Spectrum 8: FTIR spectrum of 2,6-bis(7H-acenaphtho[1,2-d]imidazol-8-yl)pyridine (3B)

FTIR (υ_{max} cm^{-1}): 3746.19(N-H), 1582.52(C-N), 1497.52(C=N)

3. ^{1}H, ^{13}C NMR, FTIR, Mass spectra of the 2,6-bis(1H-phenanthro[9,10-d]imidazol-2-yl)pyridine (3C)

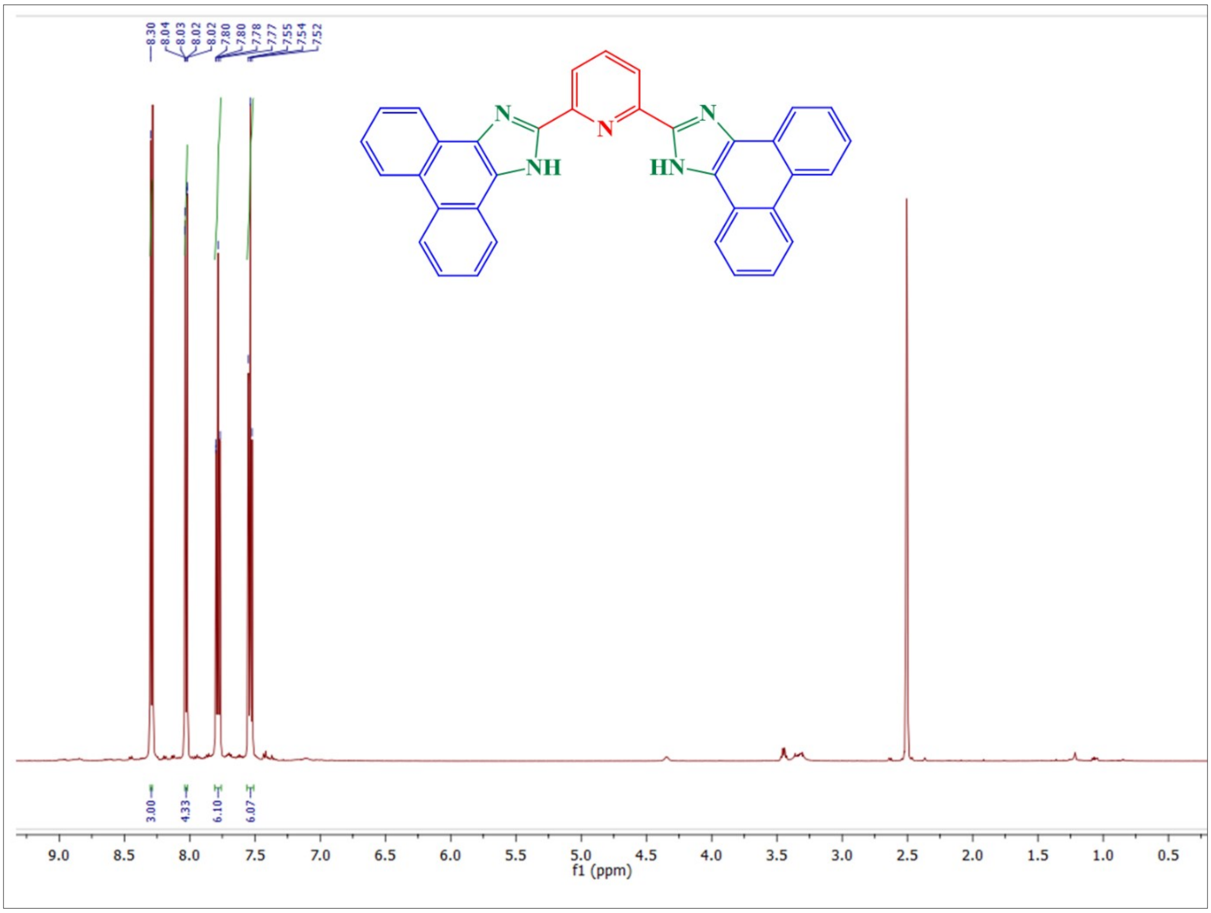

Spectrum 9: ^{1}H NMR spectrum of 2,6-bis(1H-phenanthro[9,10-d]imidazol-2-yl)pyridine (3C)

^{1}H NMR (500 MH$_{Z}$, DMSO δ ppm): 8.30 (s, 3H), 8.03-8.02 (m, 4H), 7.88-7.77 (m, 6H), 7.55-7.52 (m, 6H).

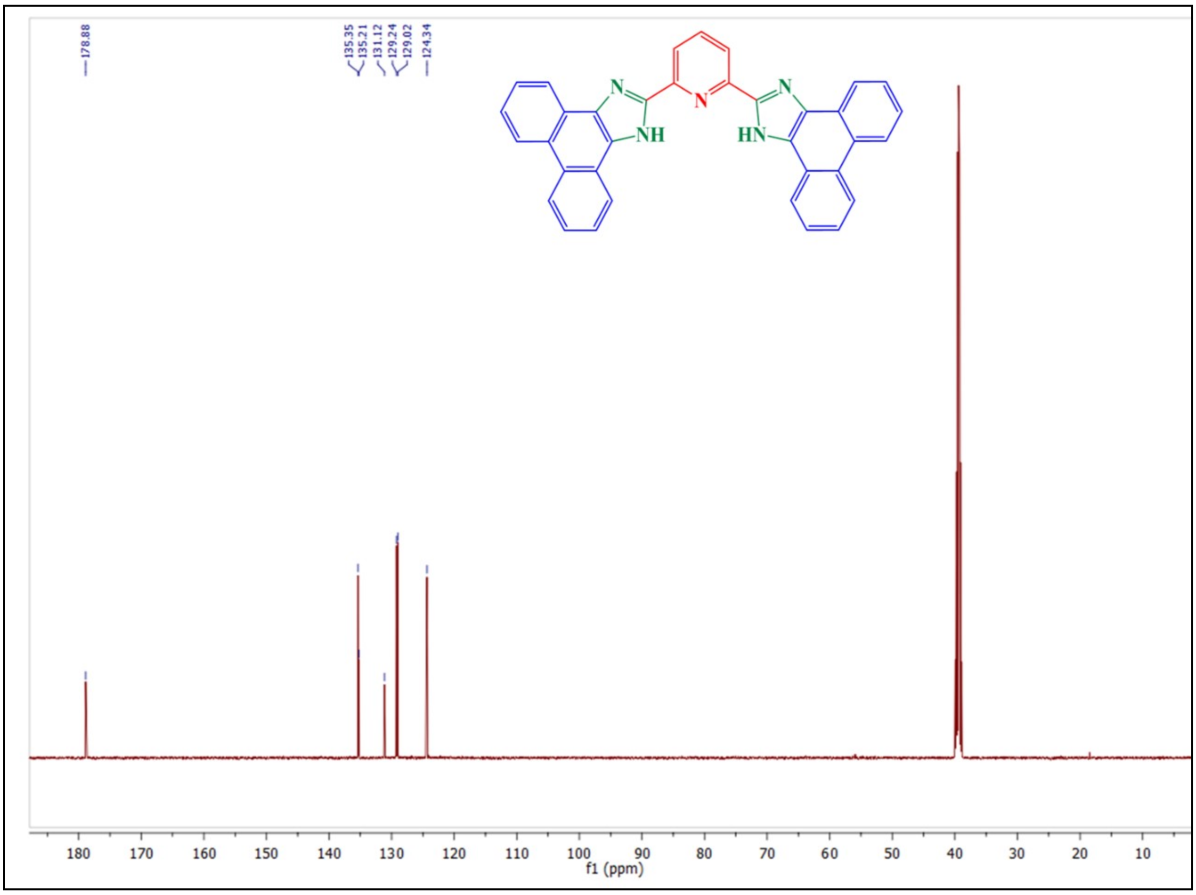

Spectrum 10: ^{13}C NMR spectrum of 2,6-bis(1H-phenanthro[9,10-d]imidazol-2-yl)pyridine (3C)

^{13}C NMR (500 MHz, DMSO δ ppm): 178.9, 135.4, 135.2, 131.1, 129.2, 129.0, 124.3.

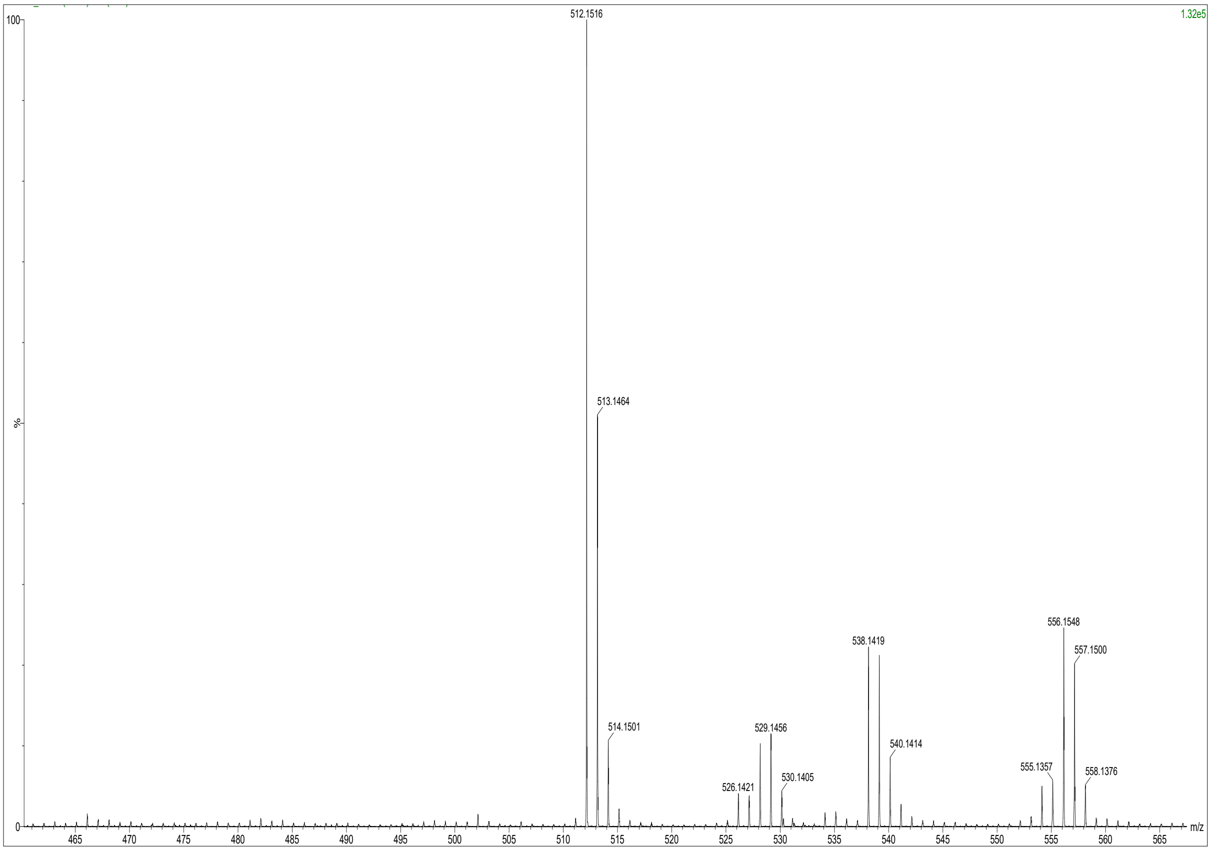

Spectrum 11: Mass spectrum of 2,6-bis(1H-phenanthro[9,10-d]imidazol-2-yl)pyridine (3C)

Mass $C_{33}H_{20}N_8O$, M^+: 512.15.

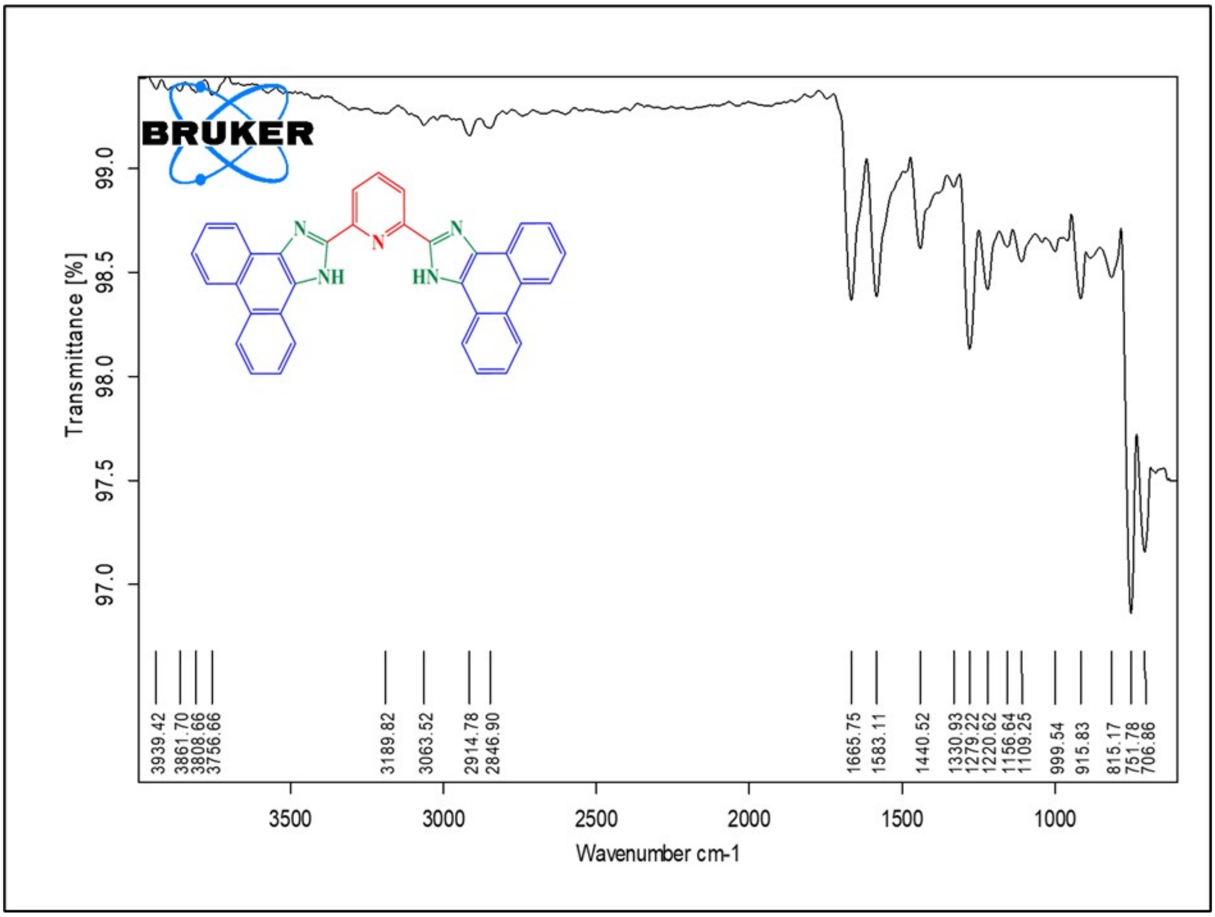

Spectrum 12: FTIR spectrum of 2,6-bis(1H-phenanthro[9,10-d]imidazol-2-yl)pyridine (3C)

FTIR (υ_{max} cm^{-1}): 3756.66 (N-H), 1583.11(C-N), 1440.52(C=N)

Density Functional Theoretical Study

Theoretical Spectrum of FTIR of synthesized Compound

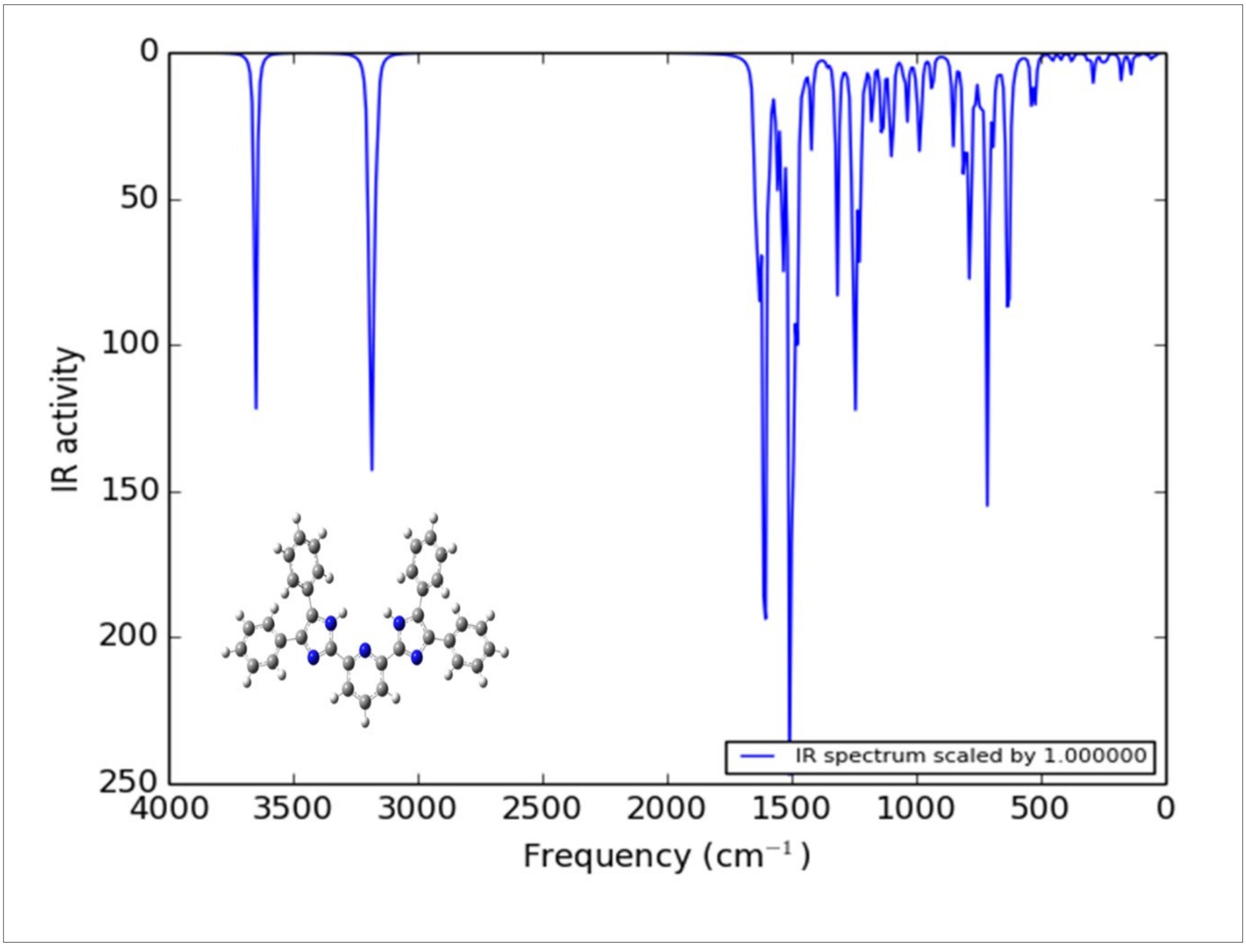

Spectrum 13: Theoretical FTIR spectrum of **2,6-bis(4,5-diphenyl-1-imidazole-2-yl) pyridine (3A)**

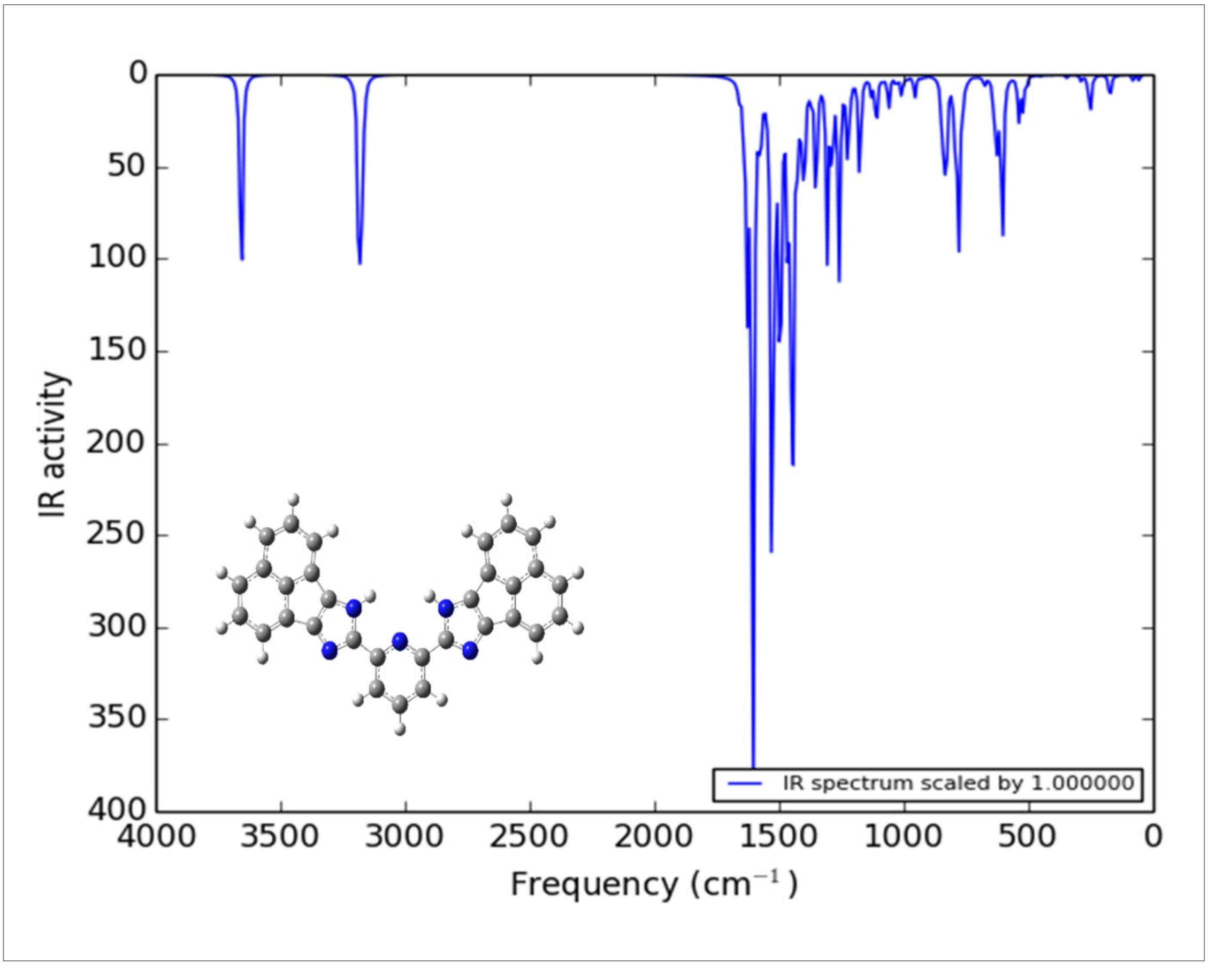

Spectrum 14: Theoretical FTIR spectrum of **2,6-bis(7H-acenaphtho[1,2-d]imidazol-8-yl)pyridine (3B)**

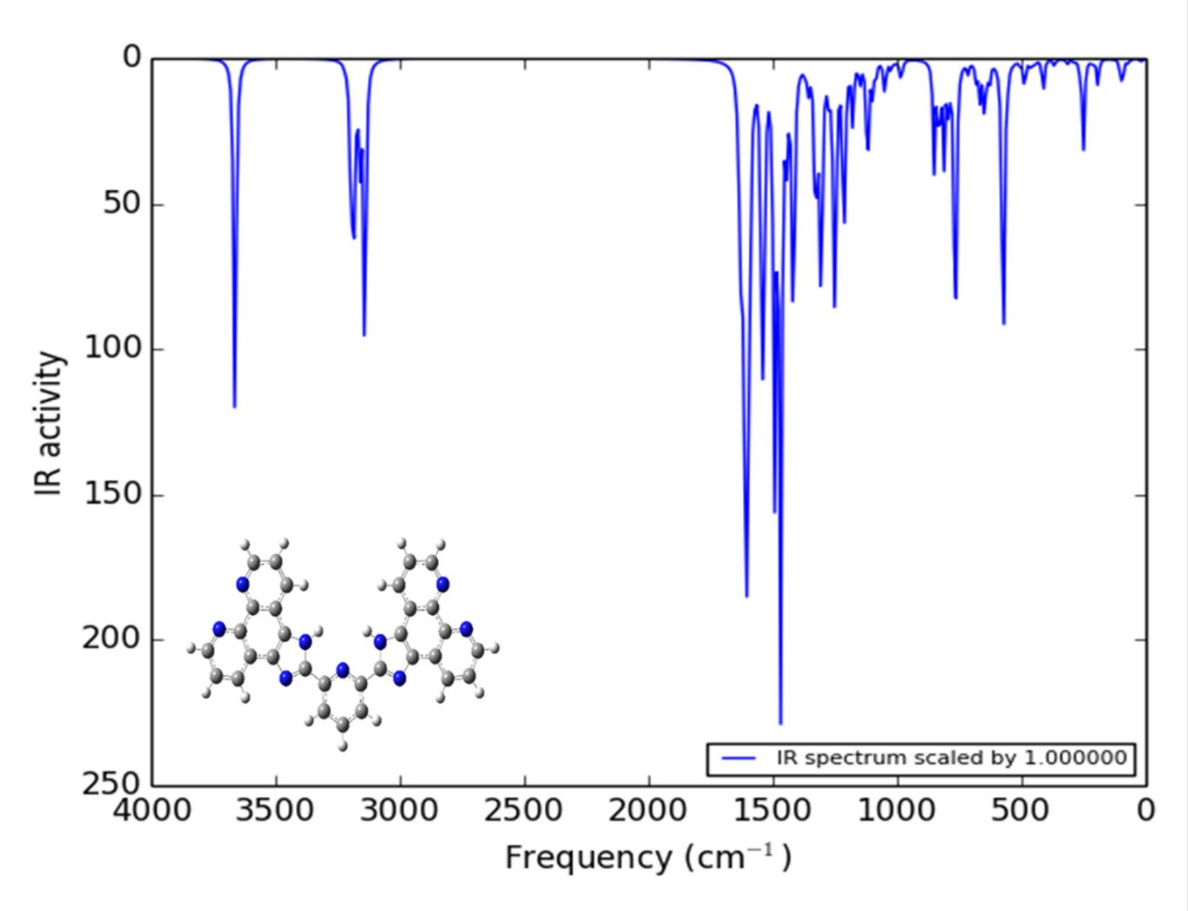

Spectrum 15: Theoretical FTIR spectrum of **2,6-bis(1H-phenanthro[9,10-d]imidazol-2-yl)pyridine (3C)**

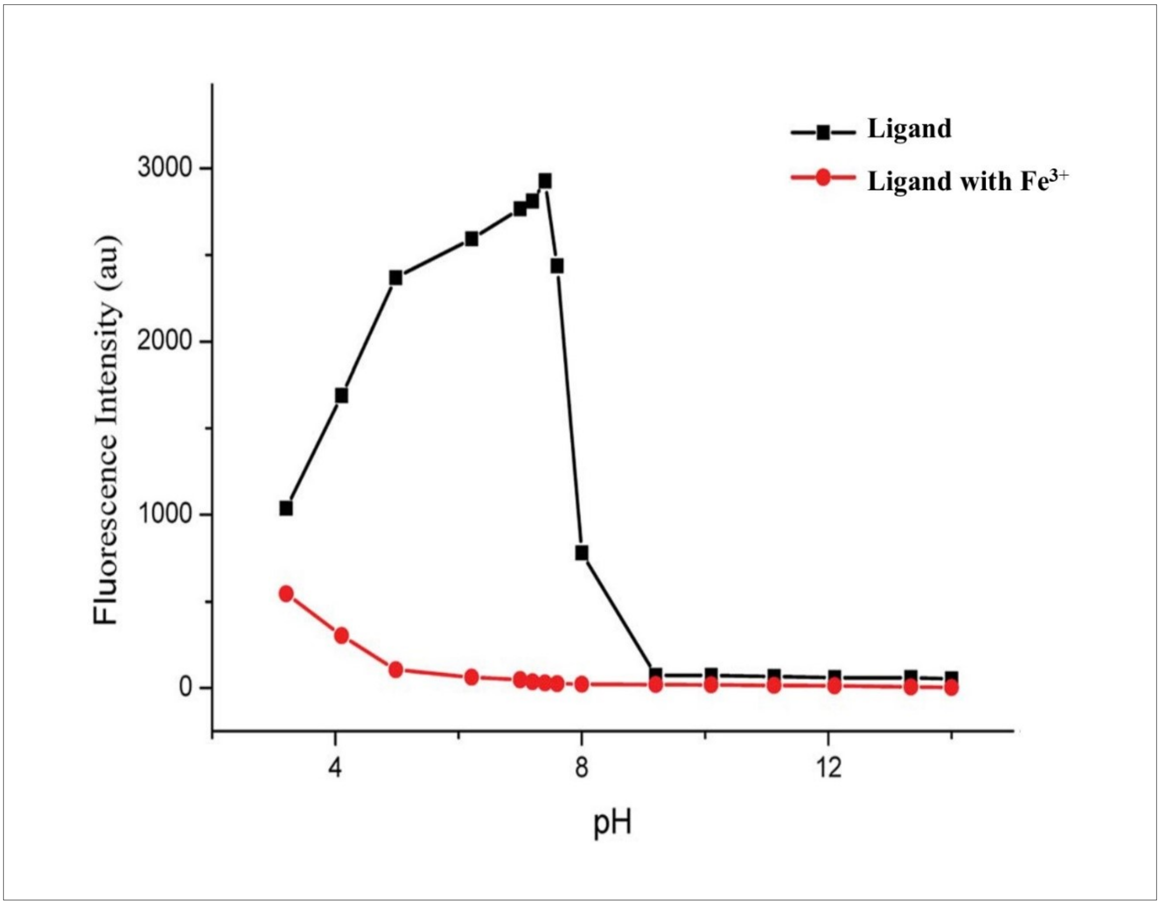

Spectrum 16: pH effect on the fluorescence intensity at 350 nm of 3A ligand (100ppm) and ligand (100ppm) with Fe^{3+} (100ppm) in ethanol

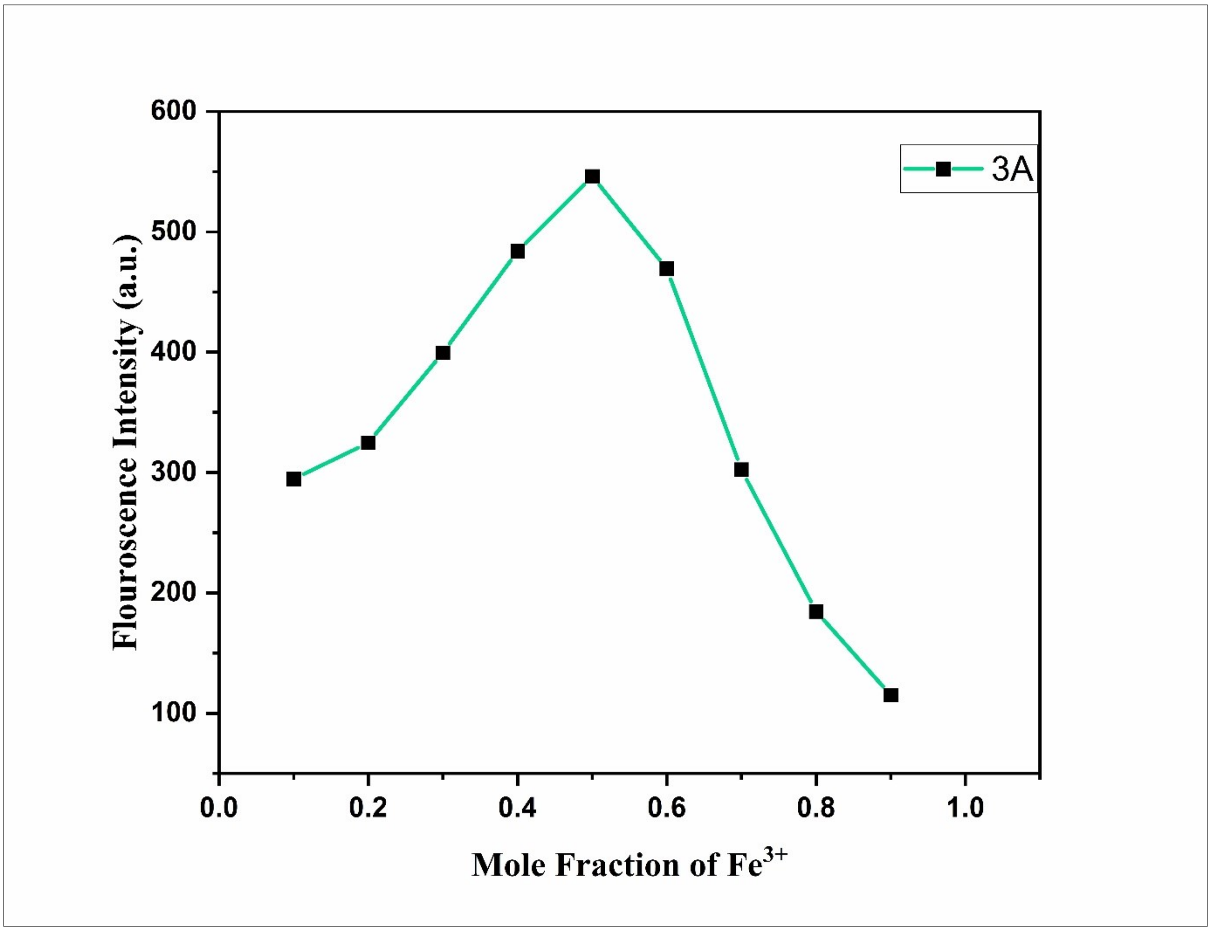

Spectrum 17: Job's plot of **2,6-bis(4,5-diphenyl-1-imidazole-2-yl) pyridine (3A)**

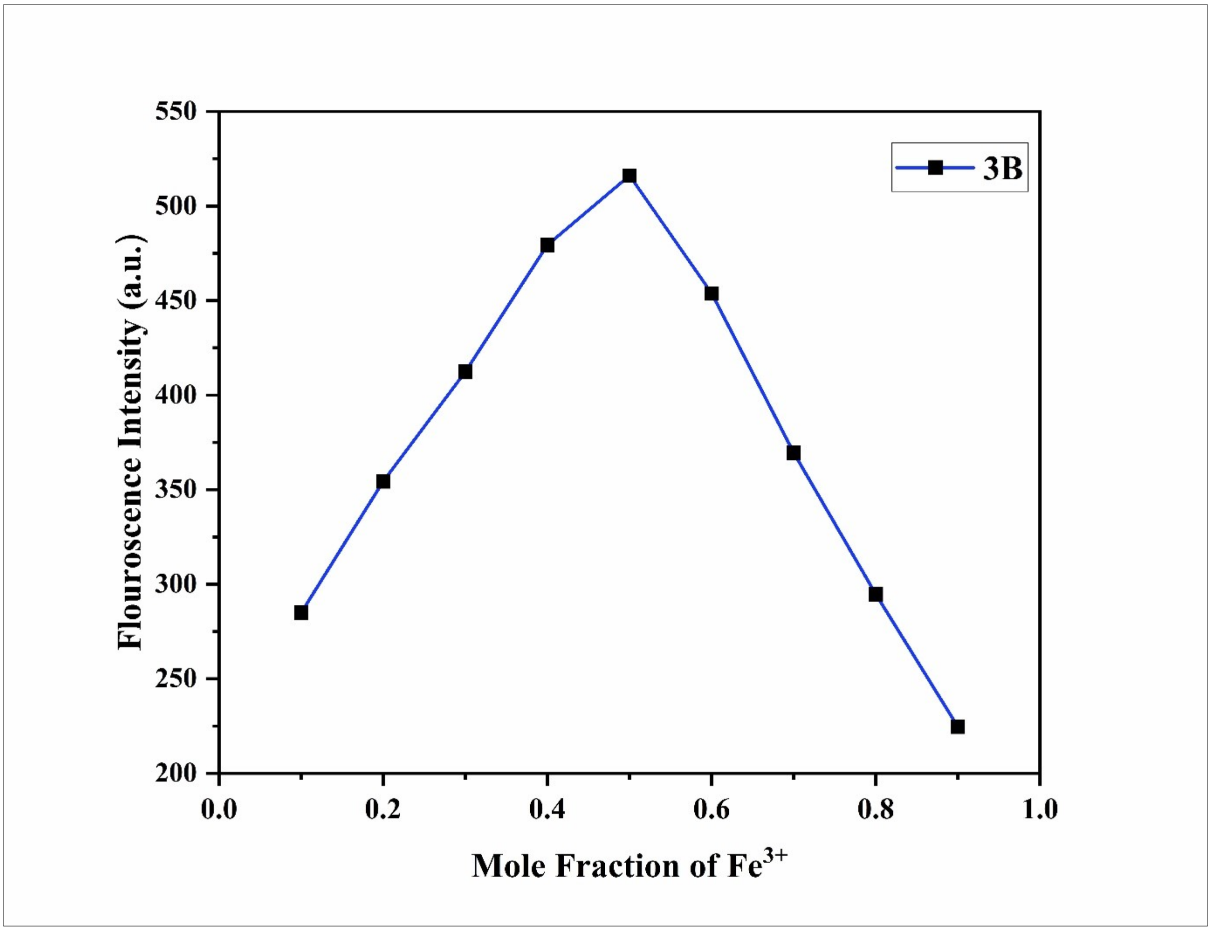

Spectrum 18: Job's plot of **2,6-bis(7H-acenaphtho[1,2-d]imidazol-8-yl)pyridine (3B)**

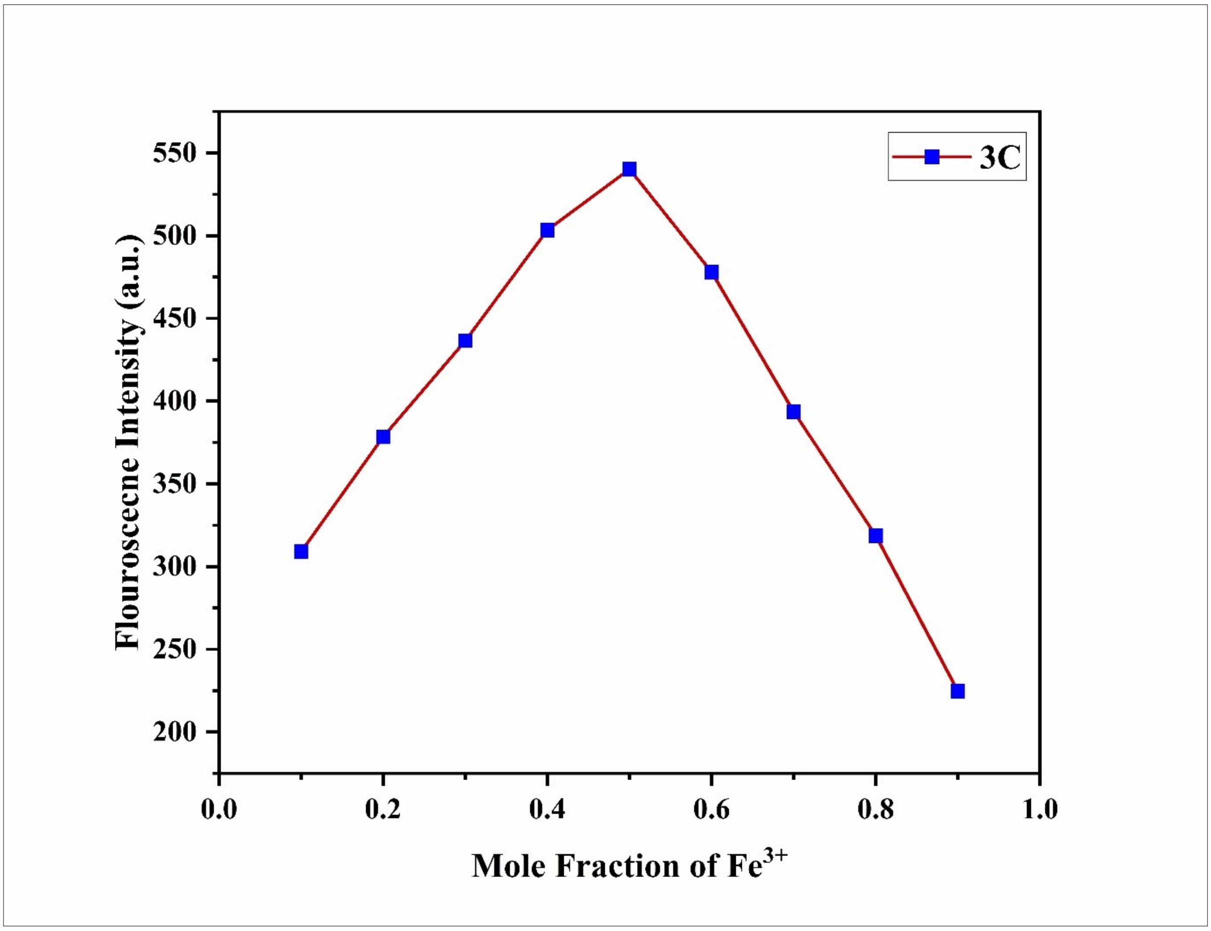

Spectrum 19: Job's plot of **2,6-bis(1H-phenanthro[9,10-d]imidazol-2-yl)pyridine (3C)**

LIST OF TABLES

Table 1: Selected geometric parameter of synthesized derivatives calculated at DFT/B3LYP/6-311G(d,p).

3A		3B		3C	
**Bond Length (Å)**		**Bond Length (Å)**		**Bond Length (Å)**	
9C-12N	1.3618	10C-50N	1.3805	47C-50N	1.3720
9C-11N	1.3195	10C-51N	1.3307	47C-51N	1.3204
12N-64H	1.0080	50N-11H	1.0073	50N-57H	1.0069
10C-13N	1.3618	13C-49H	1.3805	28C-48N	1.3720
10C-14N	1.3195	13C-48C	1.3307	28C-49N	1.3204
13N-65H	1.0080	49N-14H	1.0073	48N-56H	1.0069
5C-15N	1.3435	1C-6N	1.3446	24N-23C	1.3433
1C-15N	1.3435	5C-6N	1.3446	24N-19C	1.3433
**Bond Angle (°)**		**Bond Angle (°)**		**Bond Angle (°)**	
5C,9C,12N	122.4747	2C,1C,6N	122.9008	20C,19C,24N	122.9967
5C,9C,11N	126.7058	4C,5C,6N	122.9008	22C,23C,24N	122.9963
4C,5C,15N	122.9302	1C,10C,50N	121.6378	19C,47C,50N	121.8955
2C,1C,15N	122.9302	1C,10C,51N	126.2930	19C,47C,51N	126.1542
14N,10C,13N	110.8191	50N,10C,51N	112.0692	51N,47C,50N	111.9504
10C,13N,65H	124.1463	10C,50N,11H	123.0698	47C,50N,57H	123.4681

Table 2: Experimental and simulated IR spectra of a synthesized molecule at DFT/B3LYP/6-311G(d,p) basis set.

Assignment	3A		3B		3C	
	Experimental (cm^{-1})	Theoretical (cm^{-1})	Experimental (cm^{-1})	Theoretical (cm^{-1})	Experimental (cm^{-1})	Theoretical (cm^{-1})
**N-H**	3574.26	3648.24	3746.19	3658.21	3756.66	3661.13
**C─N**	1422.98	1448.07	1582.52	1512.86	1583.11	1575.56
**C═N**	1582.88	1590.07	1497.52	1496.10	1440.52	1437.10

Table 3. Calculation of global reactivity Parameters for titled compounds computed at DFT/B3LYP/6-311G(d,p)

**Global reactivity Descriptors**	**Compound 3A**	**Compound 3B**	**Compound 3C**
Optimization energy (Hartree)	-1622.9826	-1465.6627	-1684.7703
HOMO (eV)	-5.5092	-5.3868	-5.9805
LUMO (eV)	-1.6528	-2.1298	-2.1549
Band Gap (eV)	3.8564	3.2569	3.8257
Ionization potential (eV)	5.5092	5.3868	5.9805
Electron affinity (eV)	1.6528	2.1298	2.1549
Absolute hardness (η)	1.9282	1.6285	1.9128
Absolute softness (σ)	0.9641	0.8142	0.9564
Absolute electronegativity (χ)	3.5810	3.7583	4.0677

Table 4: Excitation energies and oscillator strengths of synthesized compound calculated at DFT/B3LYP/6-311G(d,p).

Excitation energies and oscillator strengths of 3A molecule

State	Assignment		Coefficient	Energy of transition (eV)	Wavelength (nm)	Oscillator Strength
	From	To				
S_0-S_1	HOMO	LUMO	0.70015(98.04%)	3.3359	371.66	0.3141
S_0-S_2	HOMO-1	LUMO	0.50879(51.77%)	3.6186	342.63	0.0123
	HOMO	LUMO+1	0.48543(47.12%)	3.6790	337.01	0.2605
S_0-S_3	HOMO-1	LUMO+1	0.70062(98.17%)			

Excitation energies and oscillator strengths of 3B molecule

State	Assignment		Coefficient	Energy of transition (eV)	Wavelength (nm)	Oscillator Strength
	From	To				
S_0-S_1	HOMO	LUMO	0.63066(79.54%)	2.6722	463.98	0.0645
S_0-S_2	HOMO	LUMO+1	0.59602(71.04%)	2.7176	456.22	0.0334
S_0-S_3	HOMO-1	LUMO	0.60521(73.25%)	3.1374	395.18	00
	HOMO	LUMO+1	0.35801(25.63%)			

Excitation energies and oscillator strengths of 3C molecule

State	Assignment		Coefficient	Energy of transition (eV)	Wavelength (nm)	Oscillator Strength
	From	To				
S_0-S_1	HOMO	LUMO	0.70123(98.34%)	3.3169	373.78	0.5240
S_0-S_2	HOMO-1	LUMO	0.38383(29.64%)	3.5932	345.05	0.0525
	HOMO	LUMO+1	0.58229(67.81%)			
S_0-S_3	HOMO-1	LUMO+1	0.63417(80.43%)	3.6410	340.53	0.3492
	HOMO-1	LUMO+3	0.11034(24.34%)			
	HOMO	LUMO+2	0.25695(13.20%)			

Table 5. HOMO, LUMO and Band Gap of synthesized molecule calculated at DFT/B3LYP/6-311G(d,p).

**Parameters**	**Compound 3A**	**Compound 3B**	**Compound 3C**
HOMO (eV)	-5.5092	-5.3868	-5.9805
LUMO (eV)	-1.6528	-2.1298	-2.1549
Energy Gap (eV)	3.8564	3.2569	3.8257
Dipole moment (Debye)	3.7459	3.4124	1.2903

Table 6. Electrostatic potential of targeted molecules 3A, 3B and 3C.

**Compound**	**Electrostatic potential values**
3A	-6.089e-2 to +6.089e-2
3B	-6.810e-2 to +6.810e-2
3C	-8.253e-2 to +8.253e-2

Table 7. Calculated Charge-Transfer Integral Values and Dipole Moment of titled compound

**Molecule**	t_e	t_h	**Dipole moment (Ground State)**
3A	0.0551	0.1132	3.7459
3B	0.0276	0.1512	3.4124
3C	0.0376	0.1263	1.2903

Table 8: The calculated vs. experimental λmax values and HOMO-LUMO gaps calculated at DFT/B3LYP/6-311G(d,p) basis set.

**Compound**	**Theoretical λmax**	**Experimental λmax**	**% Error**	**Band Gap**
**3A**	371.66	324	12.66%	3.8564
**3B**	463.98	373	19.43%	3.2569
**3C**	373.78	329	11.79%	3.8257

Table 9: Global reactivity parameter of metal ligand complex calculated at DFT/B3LYP/LanL2DZ basis set.

Molecules	3D	3E	3F
**Optimized Energy (Hartree)**	-1774.4996	-1617.2582	-1772.1424
**Dipole moment (D)**	1.2338	0.6349	0.9235
**HOMO (eV)**	-5.6716	-5.7570	-5.8346
**LUMO (eV)**	-4.9412	-4.9423	-5.0425
**Band gap (eV)**	0.7303	0.8147	0.7921
**Ionization energy (eV)**	5.6716	5.7570	5.8346
**Electron Affinity (eV)**	4.9412	4.9423	5.0425
**Chemical hardness (eV)**	0.3652	0.4073	0.3961
**Global Softness (eV)**	1.3692	1.2274	1.2624
**Chemical Potential (eV)**	-5.3064	-5.3497	-5.4385
**Electronegativity (eV)**	5.3064	5.3497	5.4385

LIST OF FIGURES

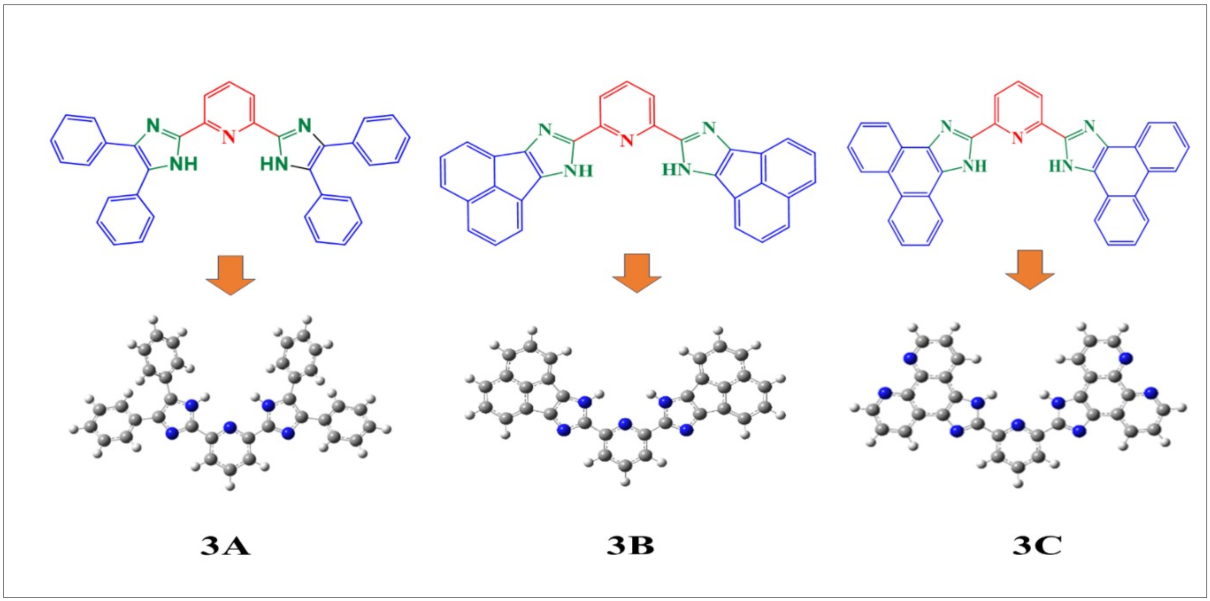

Figure 1: The optimized theoretical geometric structures of the synthesized derivates at DFT/B3LYP/6-311G(d,p)

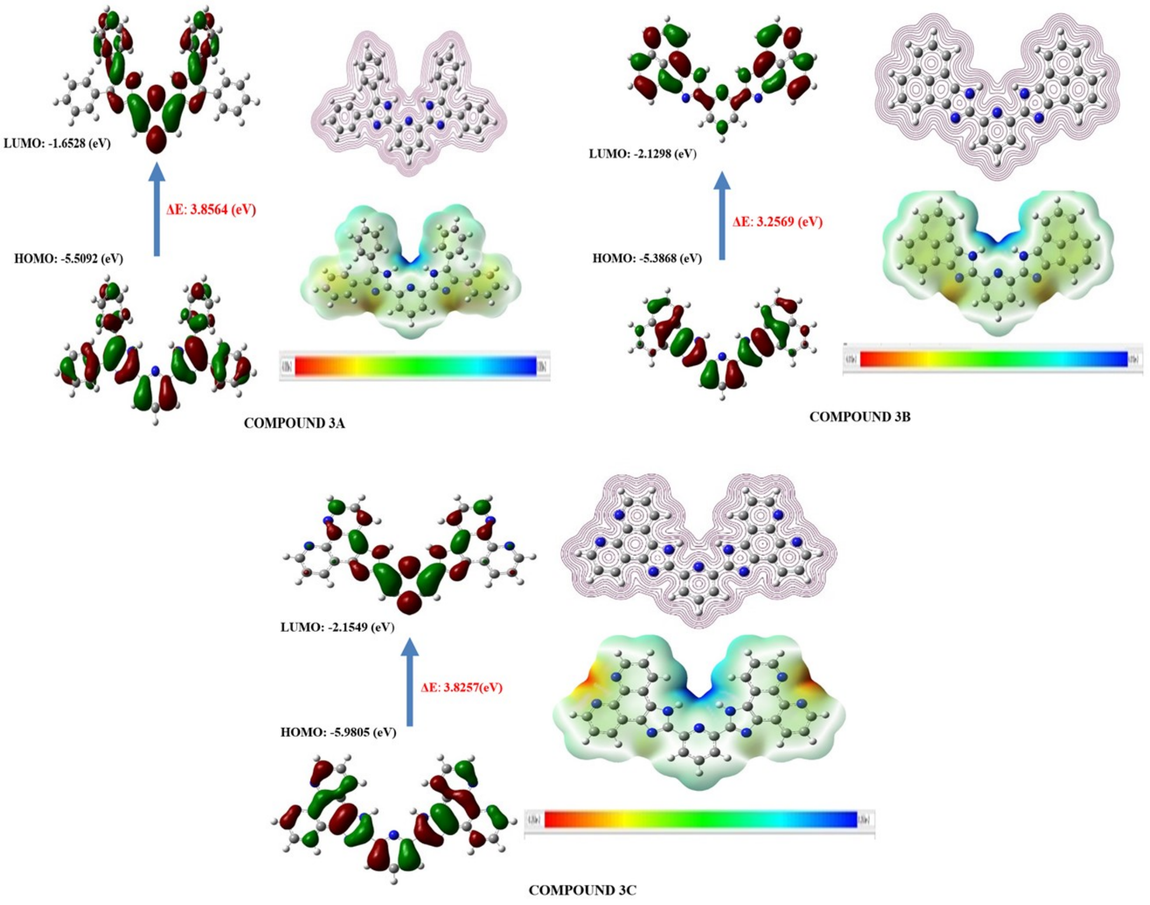

Figure 2: FMO's, molecular electrostatic potential and Contour image of 3A,3B and 3C calculated at DFT/B3LYP/6-311G(d,p).

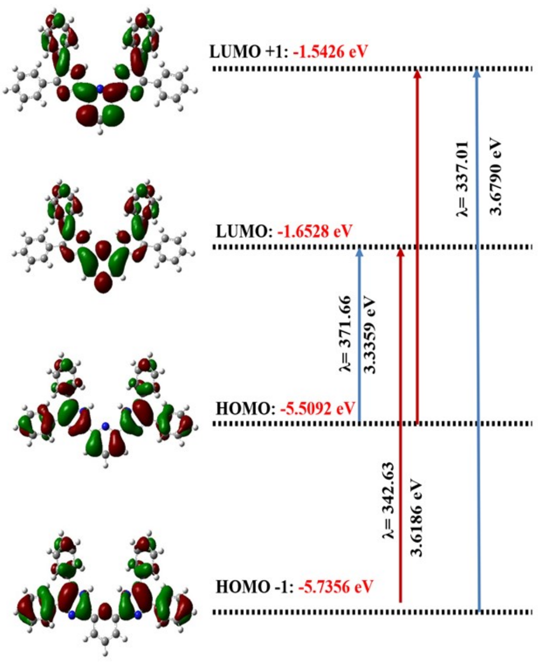

3A

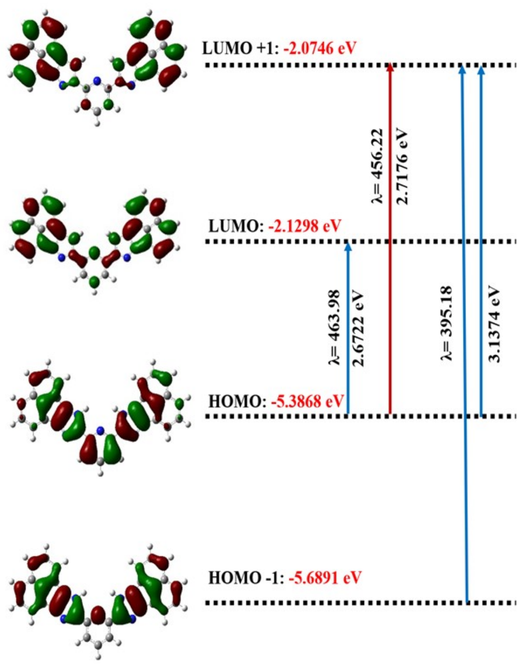

3B

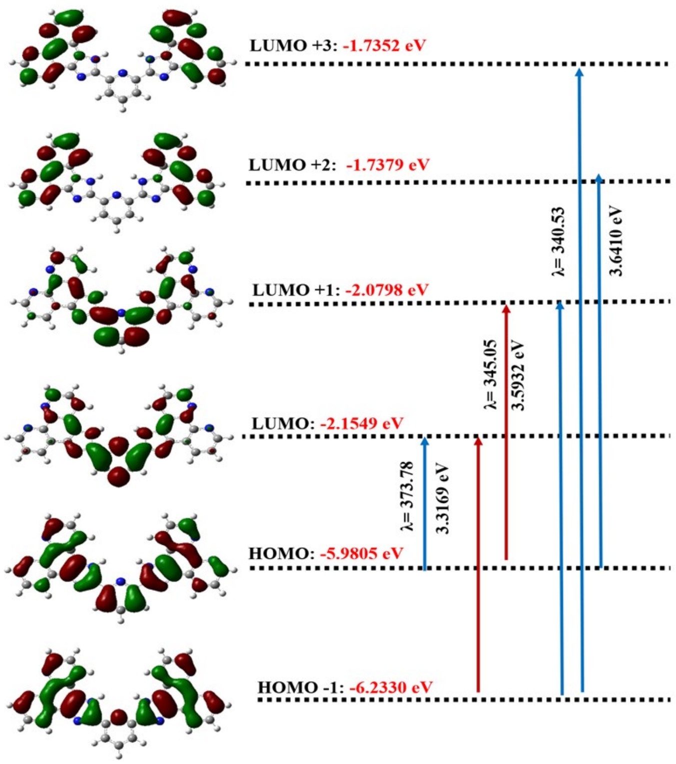

3C

Figure 3: Frontier molecular orbitals involved in the electronic absorption transitions of the compounds 3A-3C calculated at TD-DFT/B3LYP/6-311G(d,p).

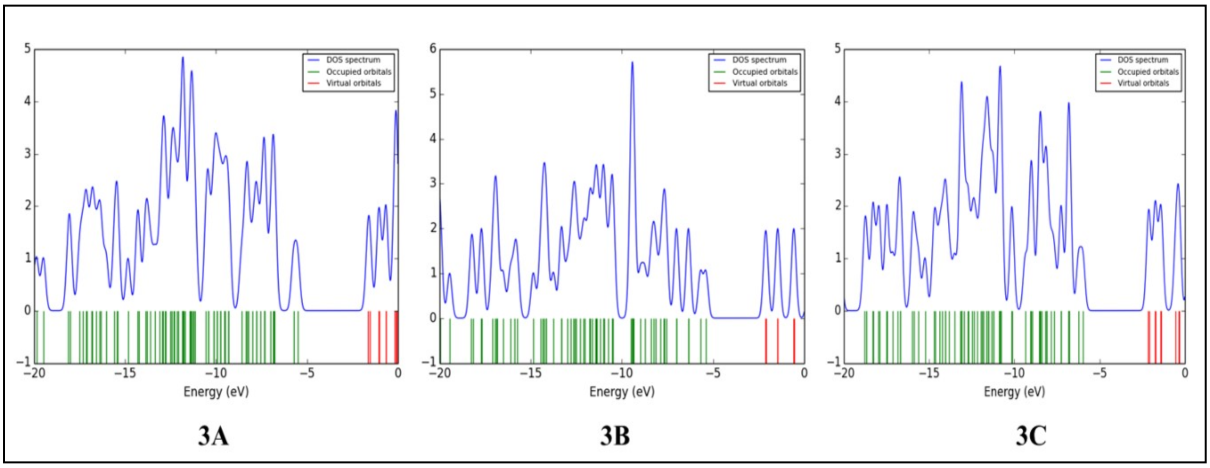

Figure 4: Density of states plot of 3A,3B and 3C calculated at DFT/B3LYP/6-311G(d,p).

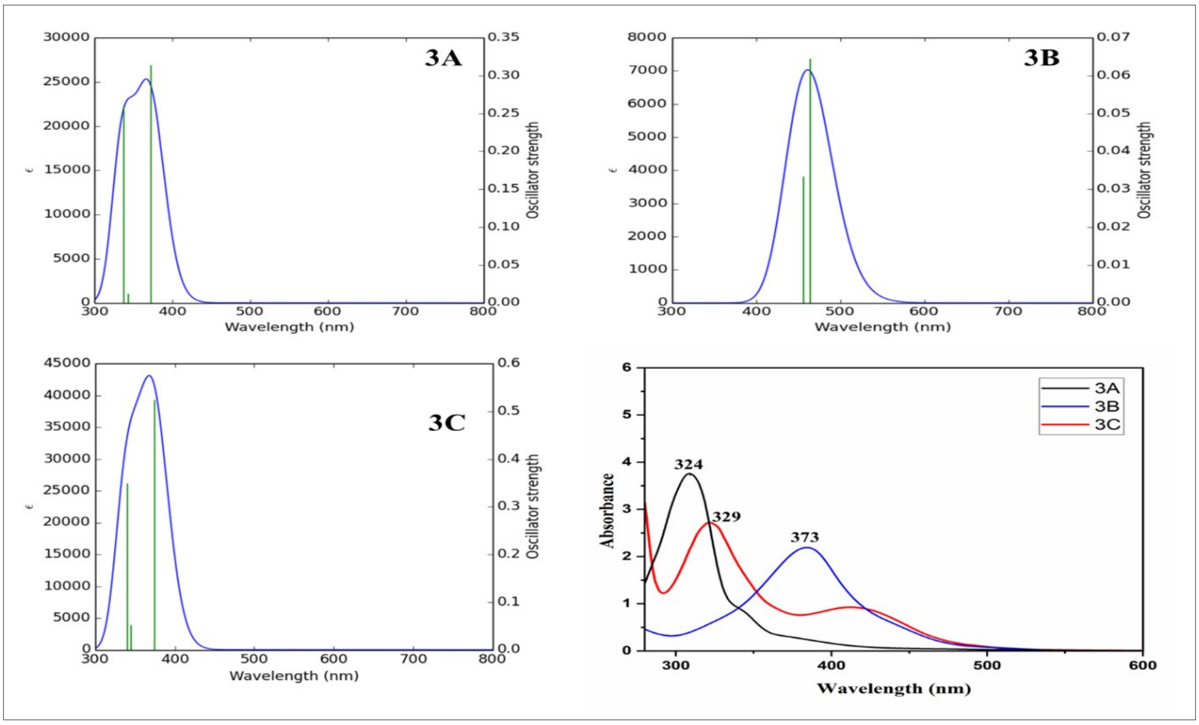

Figure 5: The experimental UV-Vis spectra and theoretical spectra calculated at DFT/B3LYP/6-311G(d,p).

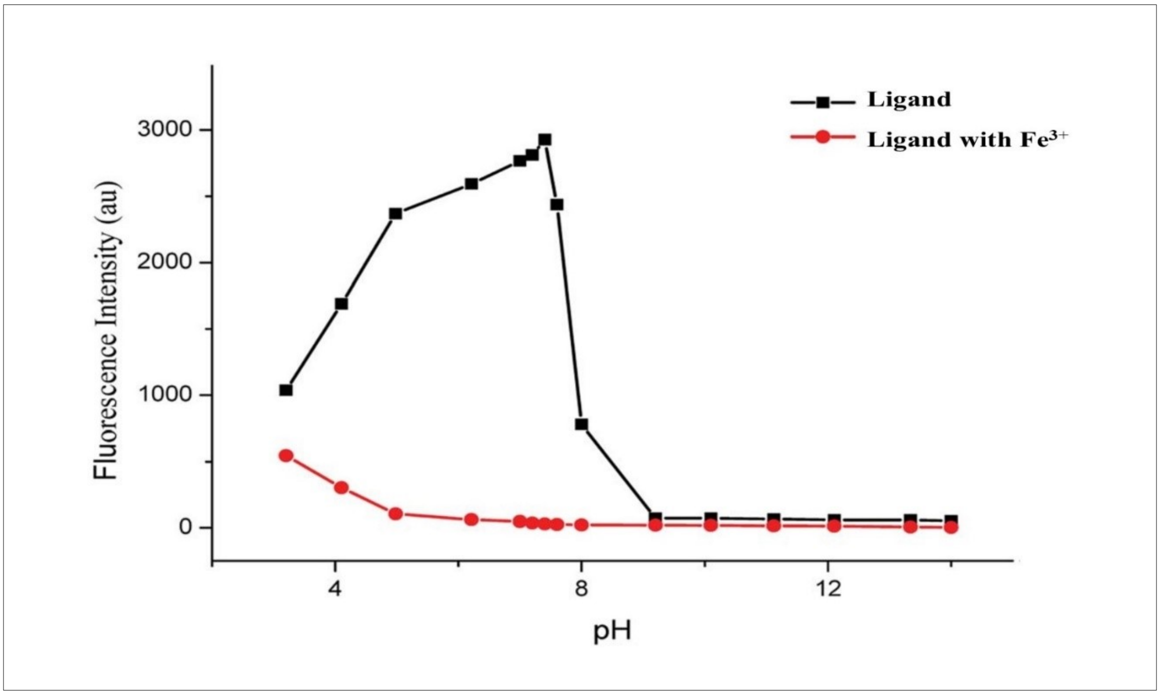

Figure 6: pH effect on the fluorescence intensity at 350 nm of 3A ligand (100ppm) and ligand (100ppm) with Fe^{3+} (100ppm) in ethanol.

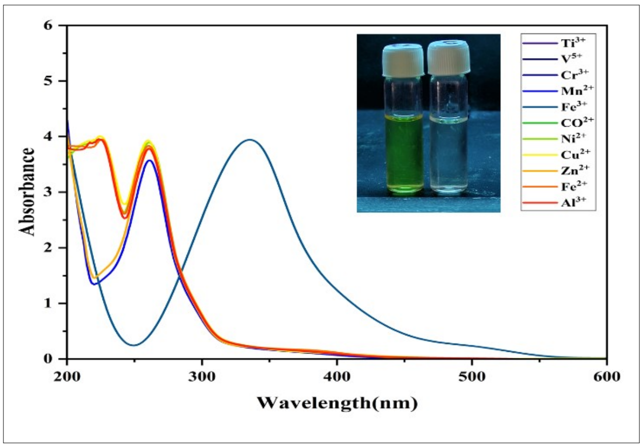

3A

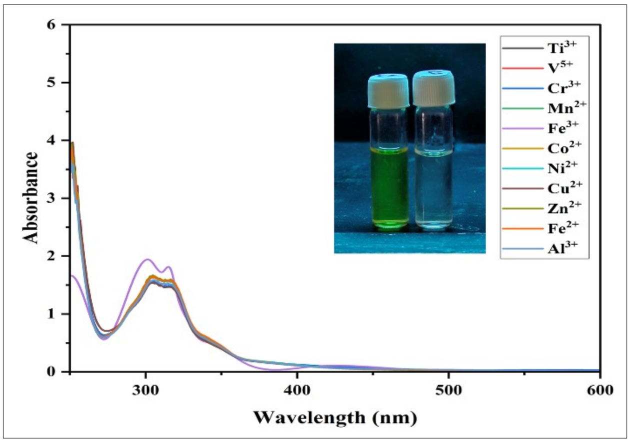

3B

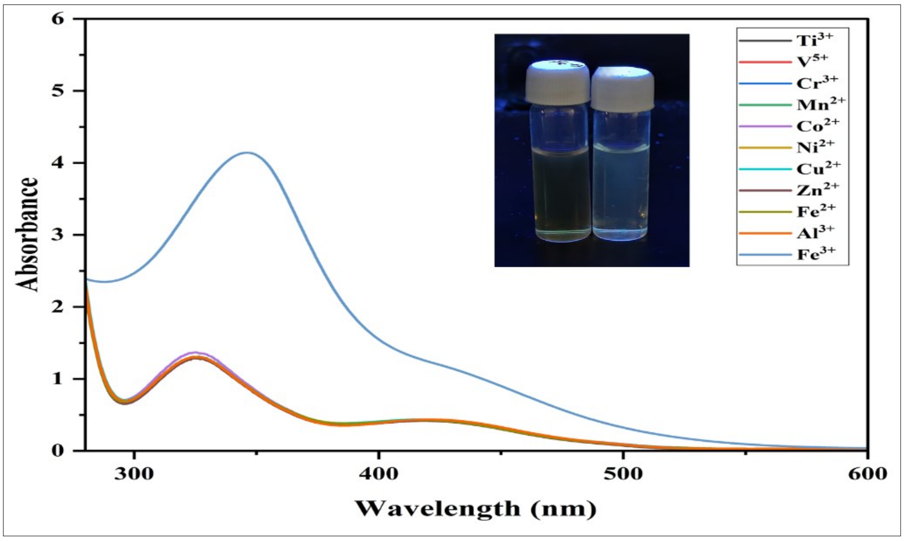

3C

Figure 7: UV-vis spectra of 3A, 3B and 3C with the addition of different metal ions (100 ppm) in ethanol at pH 7.5). Inset: colour change of the probe in the absence and presence of Fe^{3+} ions.

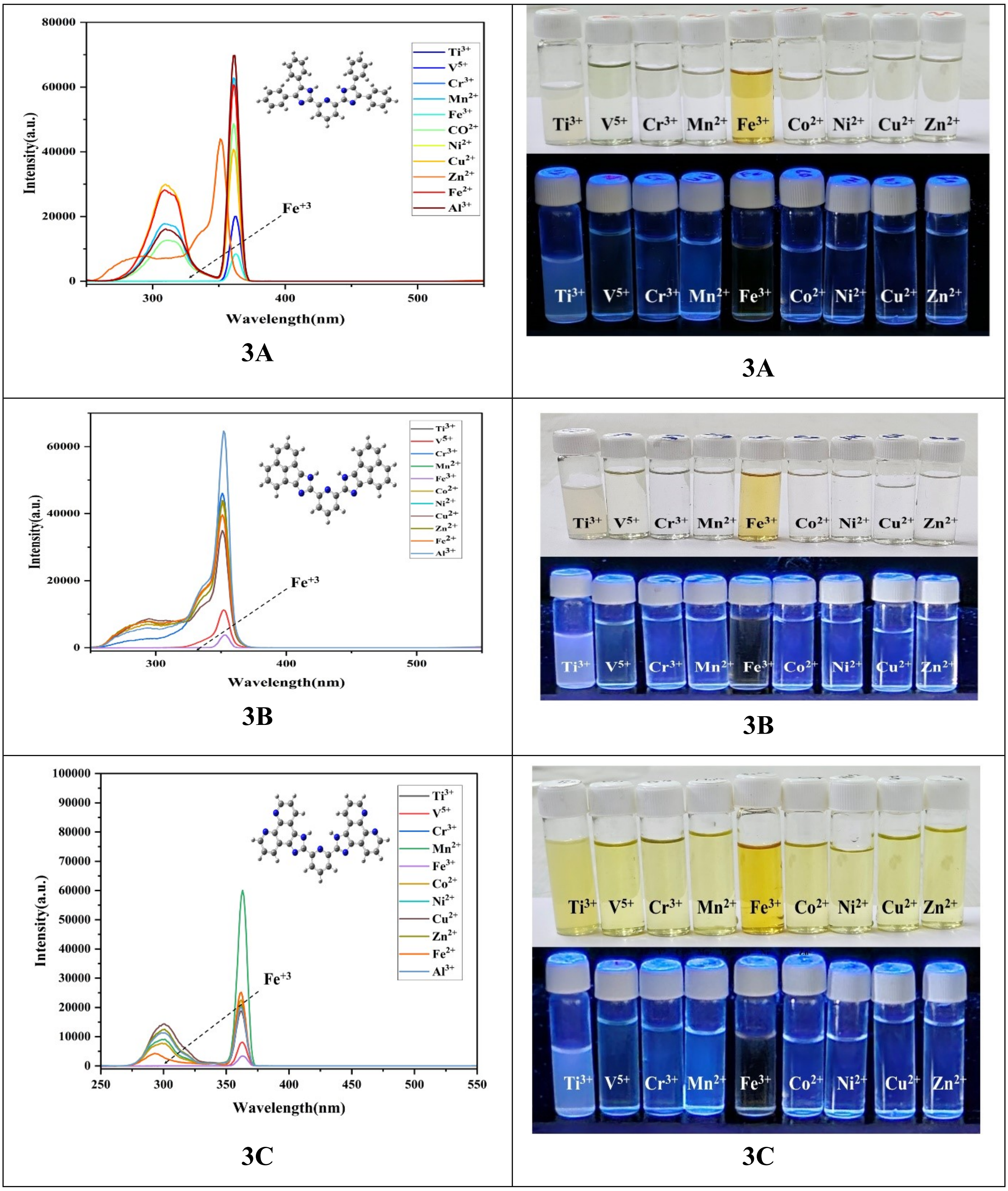

Figure 8: (a) Fluorescence spectra of all sensor 3A-3C with the adding of diverse metal ions in ethanol (pH-7.5) (b) The ligands with different metal ions (100ppm), under UV light**.**

Optimized Structure	HOMO	LUMO

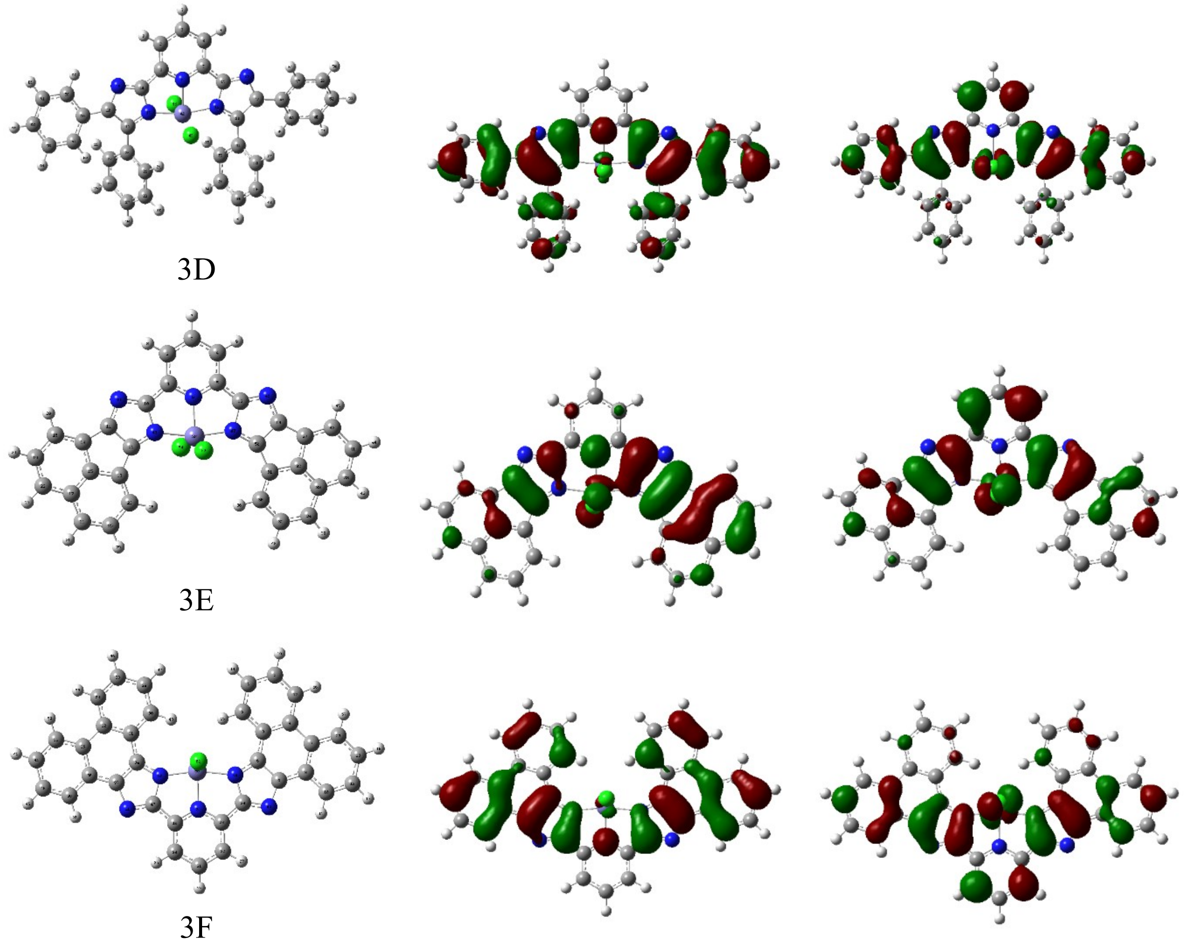

Figure 9: Optimized geometries and FMO orbital of studied proposed ferric-based complexes at the DFT/B3LYP/LanL2DZ level of theory.

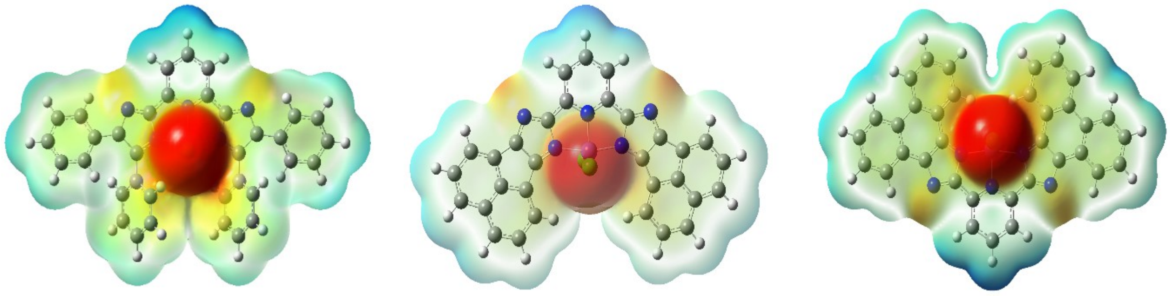

3D 3E 3F

Figure 10: MEP metal ligand complex calculated at DFT/B3LYP/LanL2DZ basis set

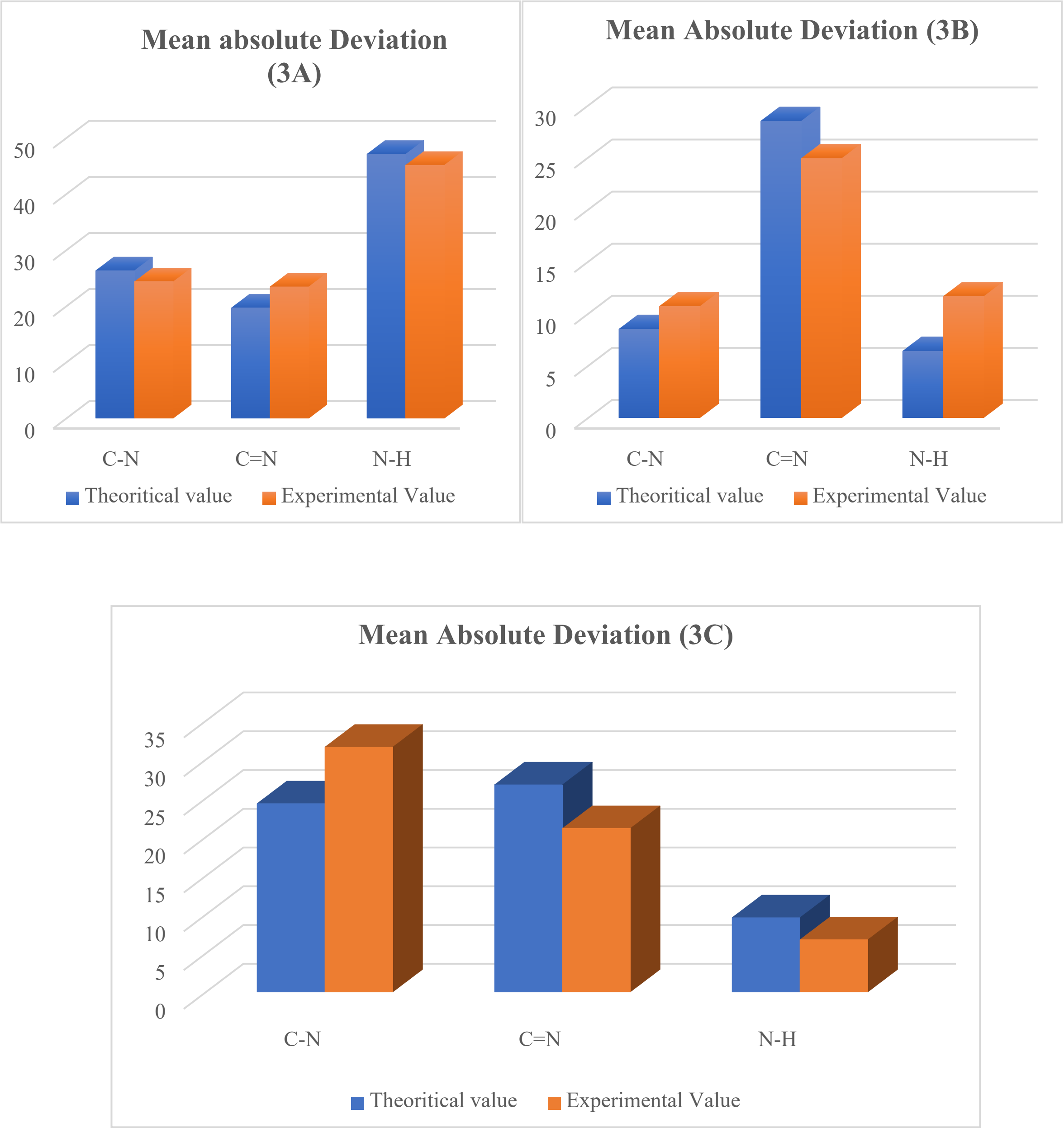

Figure 11: Mean absolute deviation of experimental and simulated IR spectra of a synthesized molecule at DFT/B3LYP/6-311G(d,p) basis set.
